# The “don’t eat me” signal CD47 is associated with microglial phagocytosis defects and autism-like behaviors in 16p11.2 deletion mice

**DOI:** 10.1073/pnas.2411080122

**Published:** 2025-04-16

**Authors:** Jun Ju, Yifan Pan, Xinyi Yang, Xuanyi Li, Jinghong Chen, Shiyu Wu, Sheng-Tao Hou

**Affiliations:** ^a^Brain Research Centre, Department of Neurobiology, School of Life Sciences, Southern University of Science and Technology, Nanshan District, Shenzhen, Guangdong 1088, People’s Republic of China

**Keywords:** autism, 16p11.2 deletion syndrome, microglia, phagocytosis, CD47

## Abstract

Various pathological characteristics of autism spectrum disorder (ASD) stem from abnormalities in brain resident immune cells, specifically microglia, to prune unnecessary synapses or neural connections during early development. Animal models of ASD exhibit an abundance of synapses in different brain regions, which is strongly linked to the appearance of ASD behaviors. Overexpression of CD47 on neurons acts as a “don’t eat me” signal, safeguarding synapses from inappropriate pruning by microglia. Indeed, CD47 overexpression occurs in 16p11.2 deletion carriers, causing decreased synaptic phagocytosis and the manifestation of ASD characteristics. However, the role of CD47 in synaptic pruning impairment leading to ASD phenotypes in the 16p11.2 deletion mouse model is unclear. Moreover, whether blocking CD47 can alleviate ASD mice’s behavioral deficits remains unknown. Here, we demonstrate a strong link between increased CD47 expression, decreased microglia phagocytosis capacity, and increased impairment in social novelty preference in the 16p11.2 deletion mice. The reduction in microglia phagocytosis caused a rise in excitatory synapses and transmission in the prefrontal cortex of 16p11.2 deletion mice. Importantly, blocking CD47 using a specific CD47 antibody or reducing CD47 expression using a specific short hairpin RNA (shRNA) enhanced the microglia phagocytosis and reduced excitatory transmission. Reduction in CD47 expression improved social novelty preference deficits in 16p11.2 mice. These findings demonstrate that CD47 is associated with the ASD phenotypes in the 16p11.2 deletion mice and could be a promising target for the development of treatment for ASD.

## Significance

Autism spectrum disorder (ASD) is a neurological developmental condition characterized by stereotyped behaviors and cognitive deficits. However, therapeutic options for ASD remain limited. Activation of the classical complement system, an innate immune signaling pathway component, supports microglia-mediated synaptic pruning during development and disease. In particular, CD47, a “don’t eat me” signal, protects synapses from inappropriate clearance. In this study, we investigated the role of CD47 in microglial phagocytosis using the 16p11.2 deletion mouse model. We found that reducing CD47 signaling enhances microglial-mediated synapse phagocytosis in the prefrontal cortex, leading to improved synaptic function and amelioration of social behavioral deficits. These findings provide mechanistic insights into the role of CD47, laying the groundwork for developing more effective treatments for ASD.

Copy number variation at the chromosomal 16p11.2 locus is strongly linked to autism spectrum disorder (ASD), a developmental neurological condition characterized by stereotyped behaviors and cognitive deficits (1–3). The 16p11.2 deletion mouse carries a deletion of the human equivalent of the 16p11.2 region and displays behavioral abnormalities similar to those found in individuals with the 16p11.2 chromosomal deletion syndrome (4, 5). Thus, the 16p11.2 deletion mouse model is a valuable tool for studying the genetic and behavioral mechanisms of ASD and serves as a platform for developing potential therapeutic interventions.

Various pathological characteristics of ASD stem from functional abnormalities of brain resident immune cells, particularly microglia (6–8). Microglia regulate synaptic circuit remodeling and phagocytose synaptic material in healthy and diseased human and mouse brains (7, 9–12). During development, microglia engulf neuronal precursors and contribute to synaptic pruning mechanisms (10). In the adult brain, microglia play a role in influencing synaptic signaling and shaping synaptic plasticity. In fact, evidence suggests that the maturation and function of distinct neural circuits may potentially be linked to the molecular identity that microglia adopt across the brain (9, 13–15). Our recent studies using the 16p11.2 deletion mouse model demonstrated a significant reduction in microglia phagocytosis, enhanced excessive excitatory neurotransmission of the pyramidal neurons in the prefrontal cortex (PFC), and their linkage with social novelty deficit (16). However, the molecular mediators for microglial regulation of synaptic steady-state in the 16p11.2 deletion mouse brain remain unclear.

Activation of the classical complement system, part of the innate immune signaling pathway, supports microglia-mediated synaptic pruning during development and disease (13–15, 17, 18). In particular, CD47, known as a “don’t eat me” signal, protects synapses from inappropriate clearance (13, 17). Overexpression of CD47 inhibits microglia engulfment in ASD (17), whereas the loss of its microglial receptor, Signal Regulatory Protein Alpha (SIRPα), enhances synaptic pruning in Alzheimer’s disease (AD) (14). CD47 is localized on active synapses, suggesting that synaptic pruning is activity-dependent and that CD47 may protect the highly active synapses from microglial pruning (13). Knockdown of CD47 or SIRPα leads to a failure in preferentially engulfing less active inputs, resulting in overpruning of synapses during postnatal development (13), in the AD brain (14), and axonal degenerative conditions (19).

The PFC is a crucial brain region regulating social behaviors and is closely associated with ASD. Individuals with 16p11.2 deletion exhibit impaired PFC connectivity with other brain regions involved in sociability functions (20). Immunohistochemical studies demonstrated elevated levels of dendritic spines in the 16p11.2 deletion mouse PFC, and electrophysiological data showed that 16p11.2 deletion mice have decreased PFC neuronal activity and abnormal NMDA receptor function (16, 21, 22). Chemogenetic activation of PFC could rescue synaptic and behavioral deficits in the mouse model of 16p11.2 deletion syndrome (22).

However, the specific role of CD47 in the 16p11.2 deletion mouse brain, particularly its impact on synaptic pruning impairment associated with ASD phenotypes, remains uninvestigated. In addition, it is also unclear whether blocking CD47 can effectively alleviate ASD behavioral deficits. In this study, we examined the influence of CD47 on microglia phagocytosis in the 16p11.2 deletion mice PFC and demonstrated that inhibiting CD47 signaling enhances microglia’s capacity to phagocytose synapses. This enhancement leads to improved synaptic function and reduced social novelty deficits.

## Results

### Impairment in Recognition Memory and Social Novelty Ability in 16p11.2 Deletion Mice.

**Fig. 1.**
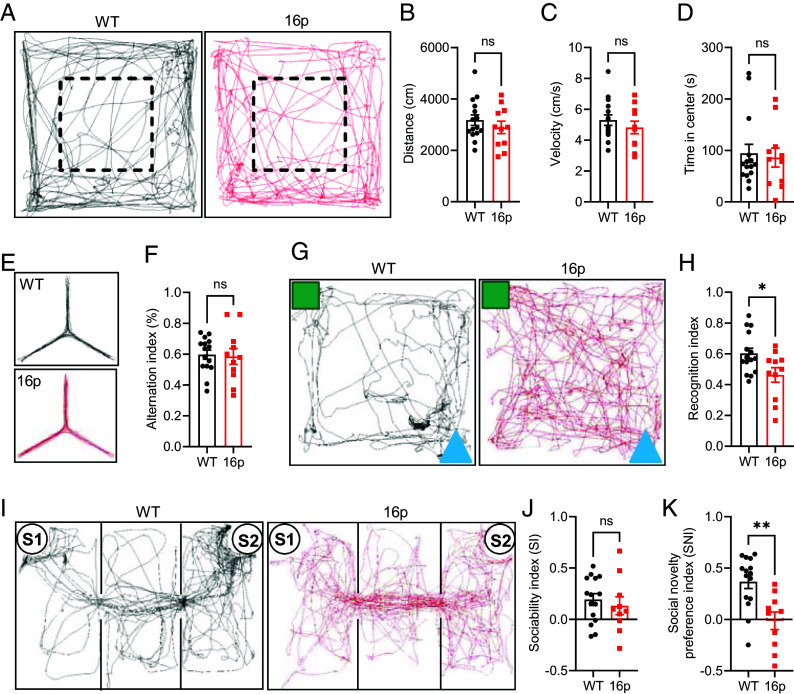
Determination of recognition memory and social novelty deficits in 16p11.2 deletion mice. (*A*) Trajectory chart of mice in the open field test, with the dashed-lined box indicating the center area. (*B*) Distance traveled by mice in the open field. (*C*) Velocity of mice in the open field. (*D*) Time spent in the center area of the open field. (*E*) Trajectory chart of mice in the Y-maze test. (*F*) Alternation index in the Y-maze test. (*G*) Trajectory chart of mice in the NOR test. Green square: familiar object; Blue triangle: novel object. (*H*) Recognition index in the NOR test. (*I*) Trajectory chart of mice in the three-chamber test. S1: familiar mouse; S2: novel mouse. (*J*) Sociability index in the three-chamber test. (*K*) Social novelty preference index in the three-chamber test. ns, not significant, *P < 0.05, **P < 0.01, unpaired *t* test for panels (*B*, *C*, *F*, *H*, and *J*) and Mann–Whitney *U* test for panels (*D* and *K*) for analysis. The numbers of mice used for experiments are shown in (*A*–*I*): *n* = 15 (WT) and *n* = 11 (16p). For panel K, WT: *n* = 15 mice, 16p: *n* = 10 mice.

Several behavioral tests were performed on the 16p11.2 deletion mice (labeled 16p in all figure panels) and the wildtype littermate (WT) controls. The open field test (OFT) was used to assess locomotion and anxiety levels. The distance traveled served as a measure of locomotion, while the time spent in the center of the open field indicated anxiety levels. In the OFT, behavioral parameters such as distance traveled, movement velocity, and time spent in the open field center showed no significant differences between 16p11.2 deletion mice and WT control mice, suggesting comparable locomotion and anxiety levels in both groups (Fig. 1 *A*–*D*). The Y-maze test was used to assess working memory. An alternation is counted when the mouse enters all three arms consecutively without revisiting any arm. The alternation index remained unchanged in the Y-maze test, indicating no differences in working memory performance between the groups (Fig. 1 *E* and *F*). The novel object recognition (NOR) test was used to assess recognition memory. The recognition index was significantly reduced in 16p11.2 deletion mice, suggesting impaired recognition memory in these animals (Fig. 1 *G* and *H*). Social behavior was assessed using a three-chamber apparatus. The 16p11.2 deletion mice exhibited significantly impaired social novelty preference, although their social ability index remained normal during the test (Fig. 1 *I*–*K*). Collectively, 16p11.2 deletion mice displayed normal locomotion, anxiety levels, and working memory, but showed deficits in recognition memory and social novelty preference.

### Reduced Microglial Phagocytosis in 16p11.2 Deletion Mice.

**Fig. 2.**
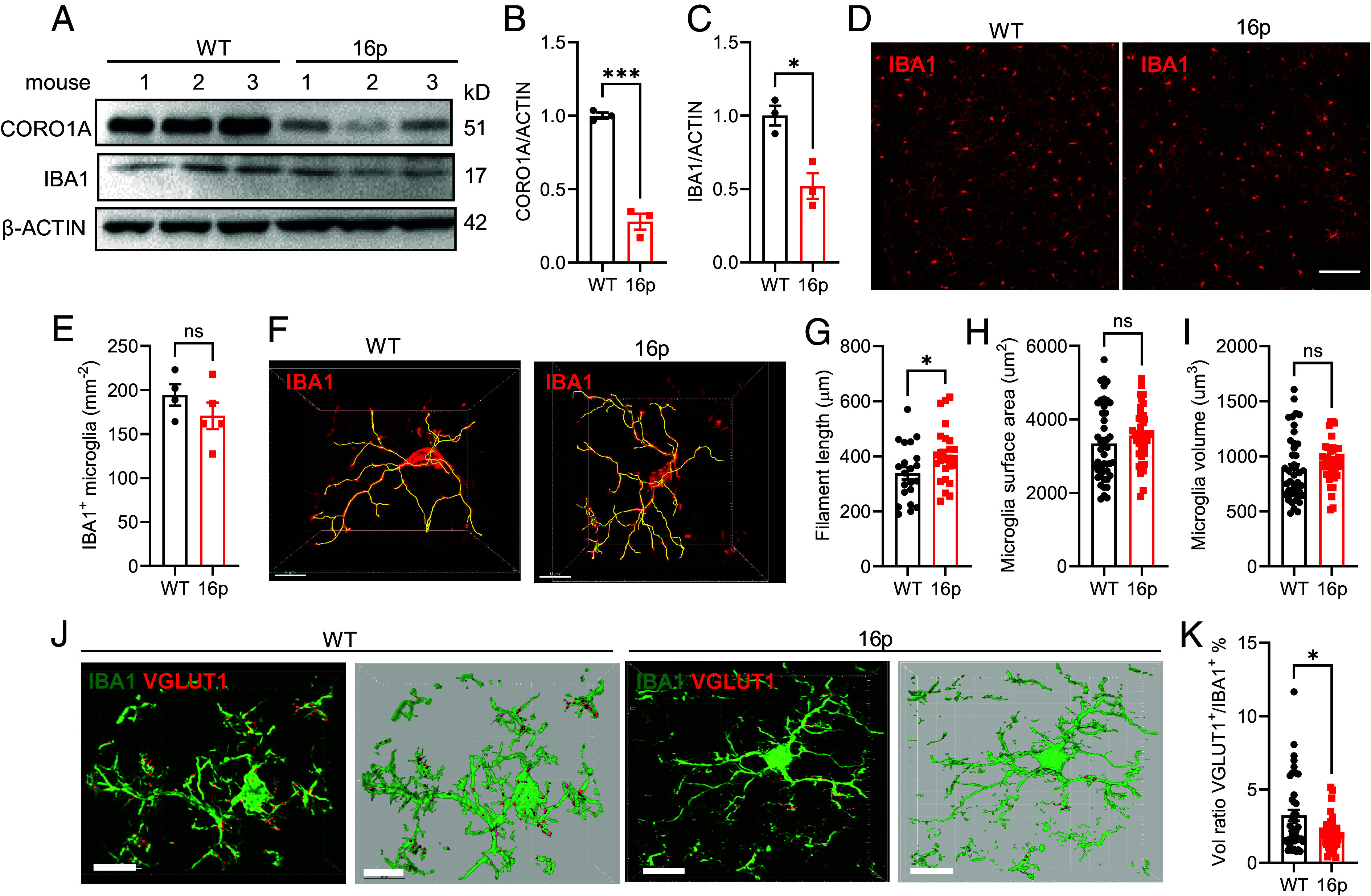
Reduction of microglia-dependent synapse pruning in 16p11.2 deletion mice. (*A*) Western blotting images showing CORO1A and IBA1 expression levels in WT and 16p group mice. β-ACTIN was used as an internal loading control. (*B*) Quantification of CORO1A expression in PFC. (*C*) Quantification of IBA1 expression in PFC (WT: *n* = 3 mice, 16p: *n* = 3 mice). (*D*) Representative images of IBA1 immunostaining in WT and 16p mice PFC. (Scale bar: 100 μm.) (*E*) Quantification of IBA1^+^ microglia density (WT: *n* = 4 mice, 16p: *n* = 5 mice). (*F*) Representative images of a single microglia. (Scale bar: 10 μm.) (*G*) Quantification of filament length of individual microglia cells (WT: *n* = 21 cells from 4 mice, 16p: *n* = 25 cells from 5 mice). (*H*) Quantification of microglia volume. (*I*) Quantification of VGLUT1 volume ratio in microglia (WT: *n* = 44 cells from 4 mice, 16p: *n* = 41 cells from 4 mice). (*J*) Representative images of IBA1 and VGLUT1 coimmunostaining in WT and 16p mice groups (*Left*: original image; *Right*: reconstructed image). (Scale bar: 10 μm.) (*K*) Quantification of microglia surface area (WT: *n* = 44 cells from 4 mice, 16p: *n* = 41 cells from 4 mice). ns, not significant, *P < 0.05, ***P < 0.001, unpaired *t* test for panels (*B*, *C*, *E*, and *G*) and Mann–Whitney *U* test for panels (*H*, *I*, and *K*).

To examine microglia alterations in 16p11.2 deletion mice, we employed western blotting to assess expressions of crucial marker proteins (Fig. 2*A*). The expression level of CORO1A, a gene located within the 16p11.2 deletion region, was significantly reduced, confirming the fidelity of the 16p11.2 deletion mouse model (Fig. 2 *A* and *B*). Furthermore, the expression level of IBA1, a microglial biomarker, was significantly reduced in the 16p11.2 deletion mice (Fig. 2 *A* and *C*). To further substantiate IBA1 change in microglia, we quantified IBA1 immunostaining density across various brain regions, including the PFC, primary motor cortex, sensory cortex, and striatum. The result revealed no significant difference in microglia density in these brain regions between the 16p11.2 deletion mice and the WT littermates (Fig. 2 *D* and *E* and *SI Appendix*, Fig. S1).

However, when the morphology of microglia from the PFC region was examined using confocal microscopy, we found a significant increase in the ramification of microglial processes in 16p11.2 deletion mice (Fig. 2 *F* and *G*), indicating microglial dysfunction. Additionally, the expression level of CD68, a marker of microglial phagocytic capacity, was decreased in the PFC of 16p11.2 deletion mice (*SI Appendix*, Fig. S2). Importantly, the microglial phagocytosis was significantly reduced as determined using IBA1 and VGLUT1 coimmunostaining. While the surface area and volume of microglia remained unchanged, the engulfed VGLUT1 in microglia was significantly reduced in 16p11.2 deletion mice compared with the WT littermates (Fig. 2 *H*–*K*). These data demonstrated reduced microglia phagocytosis capacity in 16p11.2 deletion mice. Together, these findings suggest that microglia in 16p11.2 deletion mice were at a reduced phagocytose state, which might contribute to the lack of adequate clearance of excessive synapses in the ASD brain.

Lipopolysaccharide (LPS) induces microglial phagocytosis and synaptic loss in the DG of mice (23, 24). To determine whether microglial response to LPS differs in 16p11.2 deletion mice compared to WT littermates, we injected LPS (0.2 mg/kg) for 7 d. While the body weight of 16p11.2 deletion mice was lower compared to WT littermates before LPS treatment (*SI Appendix*, Fig. S3 *A* and *B*), LPS treatment did not exacerbate this reduction in body weight in the 16p11.2 deletion mice. Interestingly, the responsiveness of microglia to LPS treatment in 16p11.2 deletion mice was similar to that in the WT mice (*SI Appendix*, Fig. S3 *C* and *D*).

### Increased Excitatory Synapse Number and Synaptic Transmission in 16p11.2 Deletion Mice.

**Fig. 3.**
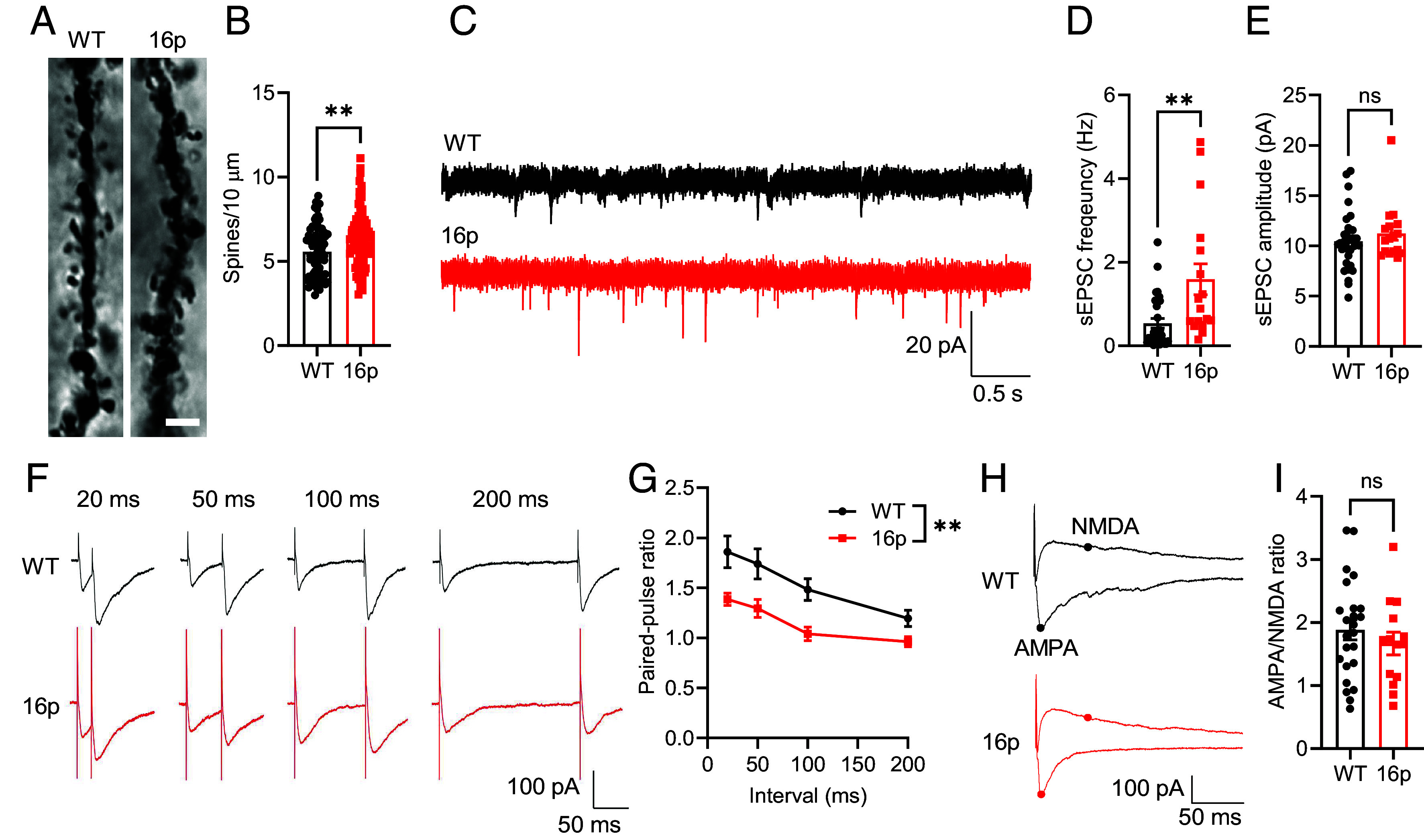
Increased excitatory synapses and excitatory transmission in 16p11.2 deletion mice. (*A*) Representative images of Golgi staining in WT and 16p mice, with scale bar: 3 μm. (*B*) Quantification of dendritic spine numbers (WT: *n* = 72 dendrites from 3 mice, 16p: *n* = 83 dendrites from 3 mice). (*C*) Representative traces of sEPSC recorded in the PFC of WT and 16p mice. (Scale bar: 20 pA/0.5 s.) (*D*) Quantification of sEPSC frequency. (*E*) Quantification of sEPSC amplitude (WT: *n* = 30 cells from 6 mice, 16p: *n* = 17 cells from 6 mice). (*F*) Representative traces of paired-pulse stimulation at intervals of 20 ms, 50 ms, 100 ms, and 200 ms in WT and 16p mice. (Scale bar: 100 pA/50 ms.) (*G*) Quantification of evoked EPSC PPR (WT: *n* = 14 cells from 4 mice, 16p: *n* = 15 cells from 6 mice). (*H*) Representative traces showing the AMPA/NMDA ratio in WT and 16p mice (α-Amino-3-Hydroxy-5-Methyl-4-Isoxazolepropionic Acid (AMPA): minimum value of the curve at −60 mV; N-Methyl-D-Aspartate (NMDA): value at 50 ms of the curve at +40 mV). (Scale bar: 100 pA/50 ms.) (*I*) Quantification of AMPA/NMDA ratio (WT: *n* = 24 cells from 6 mice, 16p: *n* = 14 cells from 6 mice). ns, not significant, **P < 0.01, unpaired *t* test for panels (*B* and *I*), Mann–Whitney *U* test for panels (*D* and *E*) and two-way RM ANOVA with Sidak’s multiple comparisons post hoc test for panel (*G*).

Experiments were designed to determine whether altered microglial phagocytosis affects the quantity and function of excitatory synapses. Using Golgi staining, we observed a significant increase in dendritic spine numbers in 16p11.2 deletion mice, indicating a defect in microglia clearance (Fig. 3 *A* and *B*). Electrophysiological measurement of spontaneous excitatory postsynaptic currents (sEPSCs) in the PFC of 16p11.2 deletion mouse showed an increased sEPSC frequency, but not amplitude, compared with the WT littermates (Fig. 3 *C*–*E*). The paired-pulse ratio (PPR) in 16p11.2 deletion mice was also reduced, indicating an elevated release probability of excitatory synapses in 16p11.2 deletion mice (Fig. 3 *F* and *G*).

However, PFC neurons in 16p11.2 deletion mice showed no changes in the ratio of AMPA/NMDA receptors (Fig. 3 *H* and *I*), nor were there any alterations in the numbers of NeuN-positive mature neurons or parvalbumin-positive (PV) interneurons in the PFC (*SI Appendix*, Fig. S4). Therefore, these data support the hypothesis that the 16p11.2 deletion mouse PFC has microglial phagocytosis defects, causing increased excitatory synapses and synaptic transmission.

### Anti-CD47 Antibody Treatment Enhanced Microglia Synaptic Pruning and Reduced Synaptic Transmission in PFC of 16p11.2 Deletion Mice.

**Fig. 4.**
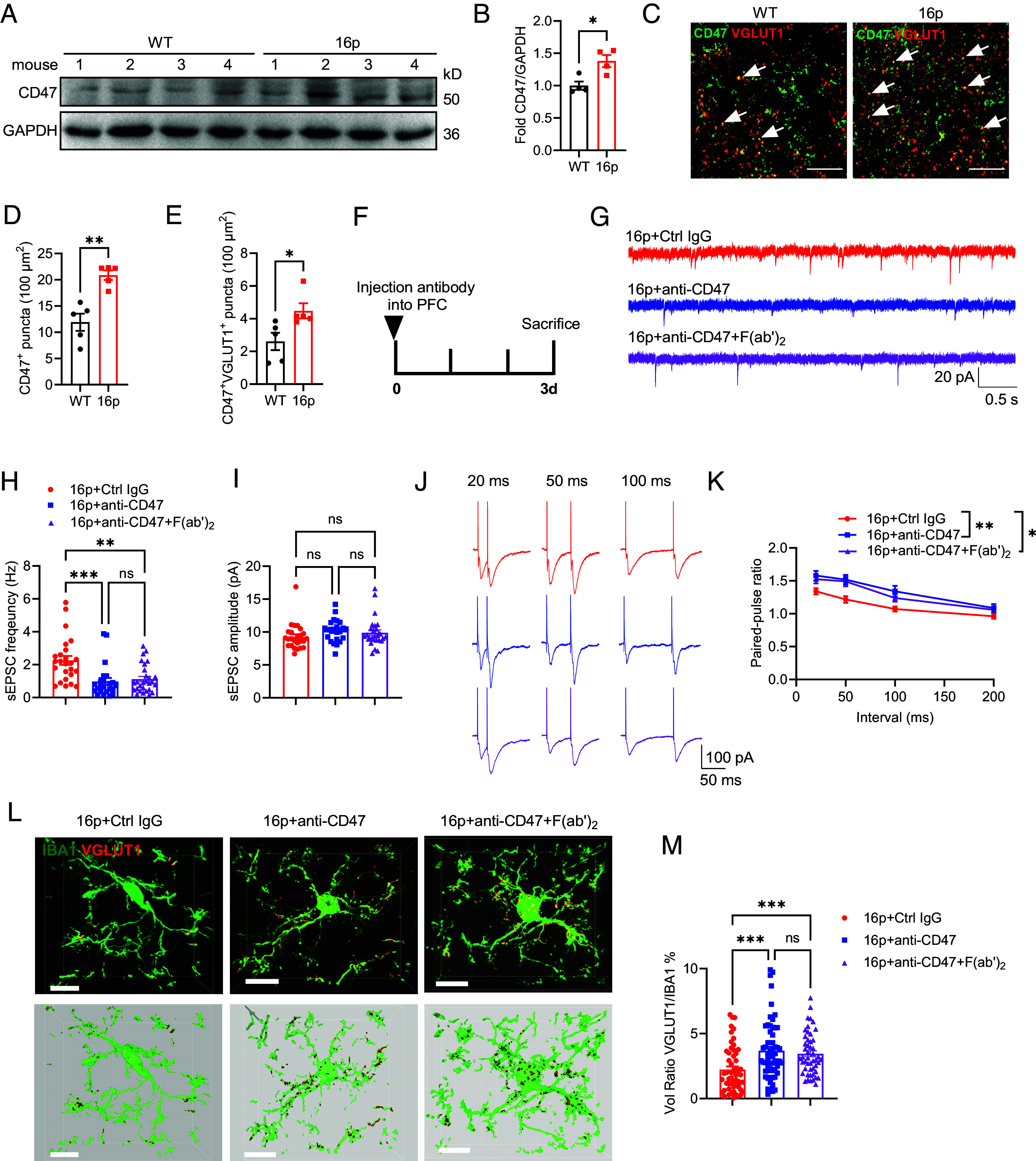
Blocking CD47 using a specific antibody in vivo reduced excitatory transmission in 16p11.2 deletion mice. (*A*) Western blot images showing CD47 expression levels in the PFC of WT and 16p mice. GAPDH was used as the internal control. (*B*) Quantification of CD47 expression in WT and 16p mice (WT: *n* = 4 mice, 16p: *n* = 4 mice). (*C*) Representative images of CD47 and VGLUT1 costaining in the PFC of WT and 16p mice. White arrows indicate CD47+VGLUT1+ puncta. (*D*) Quantification of CD47+ puncta in the PFC (WT: *n* = 5 mice, 16p: *n* = 5 mice). (*E*) Quantification of CD47+VGLUT1+ puncta in the PFC (WT: *n* = 5 mice, 16p: *n* = 5 mice). (*F*) Experimental scheme for antibody treatment. (*G*) Representative traces of sEPSCs recorded in the PFC. (Scale bar: 20 pA/0.5 s.) (*H*) Quantification of sEPSC frequency. (*I*) Quantification of sEPSC amplitude [16p+Ctrl IgG: *n* = 25 cells from 6 mice, 16p+anti-CD47: *n* = 23 cells from 6 mice, 16p+anti-CD47+F(ab’)_2_: *n* = 28 cells from 6 mice]. (*J*) Representative traces of paired-pulse stimulation at intervals of 20 ms, 50 ms, and 100 ms in two experimental groups. (Scale bar: 100 pA/50 ms.) (*K*) Quantification of PPR for evoked EPSCs. (*L*) Representative images of IBA1 and VGLUT1 coimmunostaining in the PFC. (Scale bar: 10 μm.) (*M*) Quantification of the VGLUT1/IBA1 relative volume ratios in microglia [16p+Ctrl IgG: *n* = 55 cells from 6 mice, 16p+anti-CD47: *n* = 59 cells from 6 mice, 16p+anti-CD47+F(ab’)_2_: *n* = 49 cells from 6 mice]. ns, not significant, *P < 0.05, **P < 0.01, ****P < 0.0001, unpaired *t* test for panels (*B* and *D*), Mann–Whitney *U* test for panels (*E*), two-way RM ANOVA with Sidak’s multiple comparisons post hoc test for panel (*K*) and the Kruskal–Wallis test with Dunn’s multiple comparisons post hoc test for panel (*H*, *I*, and *M*).

The neuronal expression of CD47, a negative regulator of phagocytosis, is increased in individuals with 16p11.2 deletions and is associated with brain overgrowth (17). To confirm the upregulation of CD47 expression in the brain of 16p11.2 deletion mice, we quantified CD47 protein levels using western blotting and observed a significant increase in CD47 in the PFC (Fig. 4 *A* and *B*). We further performed coimmunostaining of CD47 with NeuN, IBA1, GFAP, and OLIG2, and found that CD47 was not expressed in neuronal soma, microglia, astrocytes, or oligodendrocytes. However, CD47 was found to be expressed in synapses, where it colocalized with VGLUT1 or PSD95 (*SI Appendix*, Fig. S5). Immunostaining also confirmed the significantly increased density of CD47^+^ puncta and CD47^+^/VGLUT1^+^ puncta in the PFC of 16p11.2 deletion mice (Fig. 4 *C*–*E*). Interestingly, in contrast to human brains with the 16p11.2 deletion, the brains of 16p11.2 deletion mice showed a reduction in both weight and volume (*SI Appendix*, Fig. S6 *A*–*C*). While enhanced CD47 expression did not correlate positively with brain weight or volume, a significant negative correlation was found with the social novelty index (*SI Appendix*, Fig. S6 *D*–*F*). These findings led us to hypothesize that the increased CD47 expression in synapses might impede the phagocytic capacity of microglia.

To test this hypothesis, we investigated the in vitro and in vivo effects of blocking CD47 with a specific anti-CD47 antibody. First, brain slice preparations from 16p11.2 deletion mice were incubated with the anti-CD47 antibody in artificial cerebrospinal fluid (aCSF). Measurements of sEPSCs revealed no changes in either frequency or amplitude (*SI Appendix*, Fig. S7). Second, we stereotaxically injected the anti-CD47 antibody into the prefrontal cortex (PFC) and observed a significant reduction in sEPSC frequency in brain slice preparations 3 d postinjection, while sEPSC amplitude remained unchanged (Fig. 4 *F*–*I*). The PPR of evoked EPSCs also significantly increased (Fig. 4 *J* and *K*), suggesting a reduction in presynaptic release capacity in the 16p11.2 deletion mice following anti-CD47 treatment. Third, coimmunostaining of IBA1 and VGLUT1 showed enhanced microglial engulfment of VGLUT1 3 d after anti-CD47 treatment (Fig. 4 *L* and *M*). Fourth, the anti-CD47 antibody contains an Fc domain, which may promote synapse recognition through microglial Fc-gamma receptors. To assess the potential contribution of the Fc domain, we used F(ab’)_2_ fragments to block the Fc region of the antibody. This approach also reduced sEPSC frequency, increased the PPR of evoked EPSCs, and enhanced microglial engulfment of VGLUT1 (Fig. 4 *G*–*M*). These results demonstrate that blocking CD47 promotes synaptic pruning and reduces synaptic transmission in 16p11.2 deletion mice, independently of the Fc domain.

### Reducing CD47 Expression via shRNA Enhanced Microglia Synaptic Pruning and Diminished Synaptic Transmission in the PFC of 16p11.2 Deletion Mice.

**Fig. 5.**
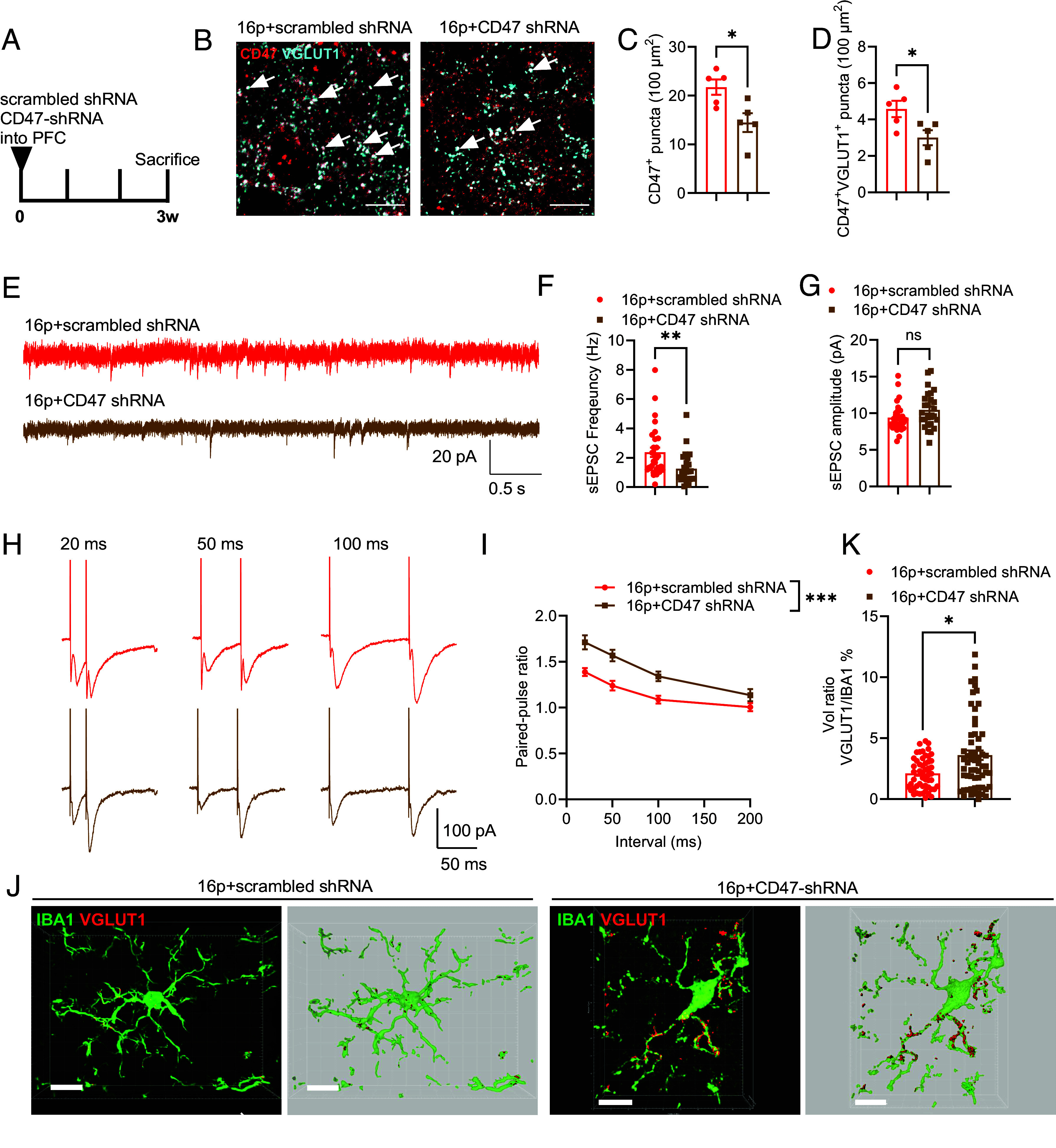
Reducing synaptic CD47 expression in vivo reduced excitatory transmission in 16p11.2 deletion mice. (*A*) Experimental scheme for stereotaxic injection of scrambled shRNA and CD47-shRNA in the PFC. (*B*) Representative images of CD47 and VGLUT1 staining in the PFC. (*C*) Quantification of CD47^+^ puncta in the PFC. (*D*) Quantification of CD47+VGLUT1^+^ puncta in the PFC (16p+scrambled shRNA: *n* = 5 mice, 16p+CD47 shRNA: *n* = 5 mice). (*E*) Representative traces of sEPSC recorded in the PFC. (Scale bar: 20 pA/0.5 s.) (*F*) Quantification of sEPSC frequency. (*G*) Quantification of sEPSC amplitude (16p+scrambled shRNA: *n* = 29 cells from 6 mice, 16p+CD47 shRNA: *n* = 26 cells from 6 mice). (*H*) Representative traces of paired-pulse stimulation at intervals of 20 ms, 50 ms, and 100 ms. (Scale bar: 100 pA/50 ms.) (*I*) Quantification of PPR for evoked EPSCs (16p+scrambled shRNA: *n* = 17 cells from 6 mice, 16p+CD47 shRNA: *n* = 20 cells from 6 mice). (*J*) Representative images of IBA1 and VGLUT1 coimmunostaining in the PFC. (Scale bar: 10 μm.) (*K*) Quantification of VGLUT1/IBA1 volume ratios in microglia (16p+scrambled shRNA: *n* = 52 cells from 5 mice, 16p+CD47 shRNA: *n* = 60 cells from 5 mice). ns, not significant; *P < 0.05, **P < 0.01, ***P < 0.001, unpaired *t* test for panels (*C* and *D*), Mann–Whitney *U* test for panel (*F*, *G*, and *K*) and two-way RM ANOVA with Sidak’s multiple comparisons post hoc test for panel (*I*).

To further investigate the impact of CD47 on PFC microglial synaptic pruning and synaptic transmission, a recombinant adeno-associated virus (rAAV) carrying CD47 shRNA (denoted as CD47-shRNA in figure panels) was stereotaxically injected into the PFC. Due to the hSyn promoter, over 90% of ZsGreen positive cells were colabeled with NeuN after 3 wk (*SI Appendix*, Fig. S8). Immunostaining revealed a reduction in the density of CD47^+^ puncta and CD47^+^/VGLUT1^+^ puncta in the PFC of 16p11.2 deletion mice following rAAV CD47-shRNA treatment (Fig. 5 *A*–*D*). Electrophysiological recordings showed a decrease in sEPSC frequency, while the sEPSC amplitude remained unchanged after treatment (Fig. 5 *E*–*G*). The PPR of evoked EPSCs was increased, indicating a reduced presynaptic release capacity in these mice (Fig. 5 *H* and *I*). Importantly, coimmunostaining of IBA1 and VGLUT1 revealed enhanced engulfment of VGLUT1 by microglia following rAAV CD47-shRNA treatment (Fig. 5 *J* and *K*).

### rAAV CD47-shRNA Treatment Enhanced Social Novelty Ability in 16p11.2 Deletion Mice.

**Fig. 6.**
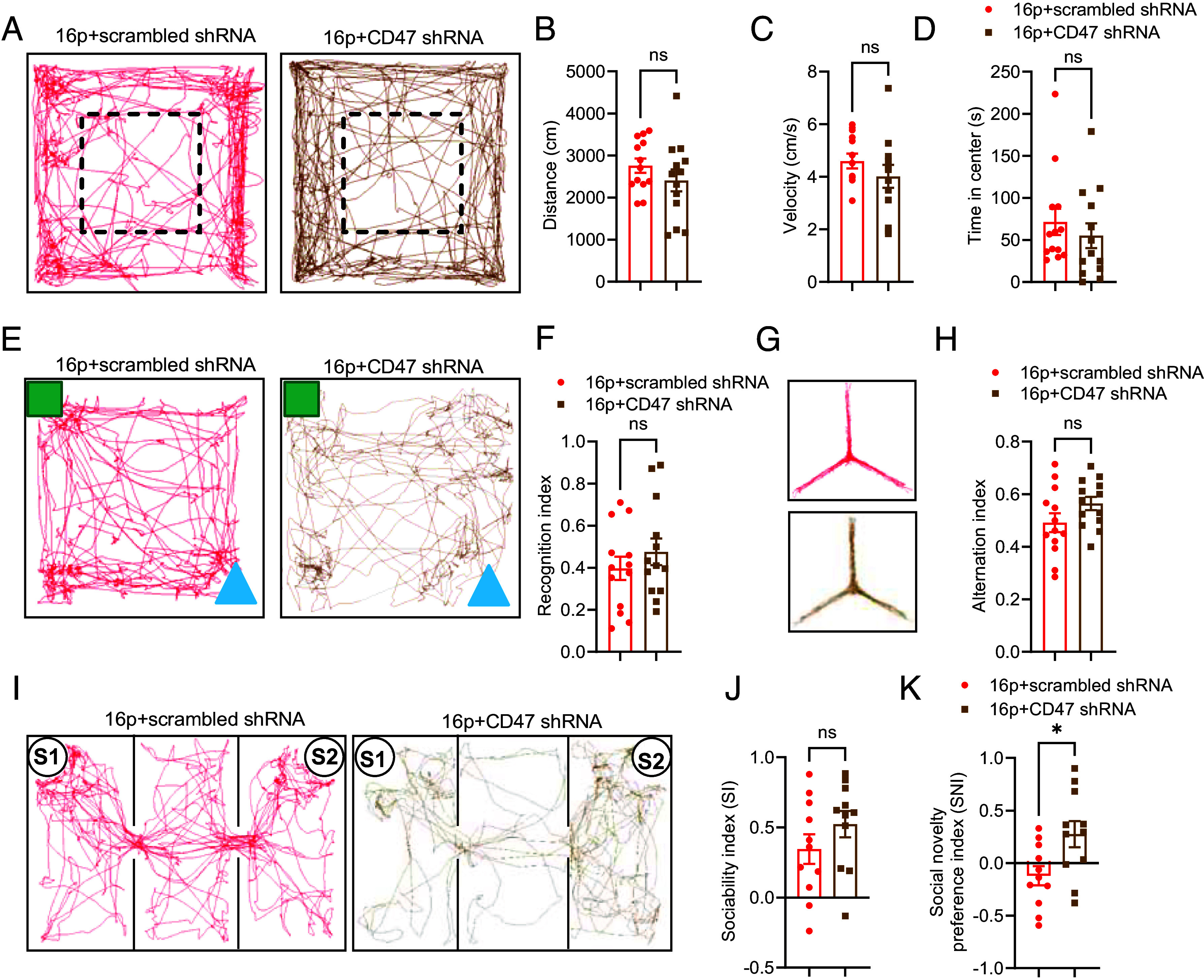
Reducing synaptic CD47 expression enhanced social novelty ability in 16p11.2 deletion mice. (*A*) Trajectory chart of mice in the open field. The dashed box represents the center area. (*B*) Distance traveled by mice in the open field. (*C*) Movement velocity of mice in the open field. (*D*) Time spent in the center of the open field (16p+scrambled shRNA: *n* = 13 mice, 16p+CD47 shRNA: *n* = 13 mice). (*E*) Trajectory chart of mice in the NOR test. Green square: familiar object; Blue triangle: novel object. (*F*) Recognition index in the NOR test (16p+scrambled shRNA: *n* = 13 mice, 16p+CD47 shRNA: *n* = 13 mice). (*G*) Trajectory chart of mice in the Y-maze test. (*H*) Alternation index in the Y-maze test (16p+scrambled shRNA: *n* = 13 mice, 16p+CD47 shRNA: *n* = 13 mice). (*I*) Trajectory chart of mice in the three-chamber sociability test. S1: familiar mouse; S2: novel mouse. (*J*) Sociability index in the three-chamber test. (*K*) Social novelty preference index in the three-chamber test (16p+scrambled shRNA: *n* = 11 mice, 16p+CD47 shRNA: *n* = 11 mice). ns, not significant, *P < 0.05, unpaired *t* test for panels (*B*, *C*, *F*, *H*, *J*, and *K*) and Mann–Whitney *U* test for panel (*D*).

Several behavioral tests were conducted on 16p11.2 deletion mice treated with rAAV CD47-shRNA to assess the potential benefits of modulating CD47. In the OFT, rAAV CD47-shRNA treatment had no significant effect on distance traveled, velocity, or time spent in the center, indicating normal locomotion and anxiety levels (Fig. 6 *A*–*D*). Similarly, the recognition index in the NOR test remained unchanged, suggesting that rAAV CD47-shRNA treatment did not improve impaired recognition memory (Fig. 6 *E* and *F*). In the Y-maze test, the alternation index also showed no significant change (Fig. 6 *G* and *H*). However, the three-chamber test revealed that rAAV CD47-shRNA treatment significantly enhanced social novelty preference (Fig. 6 *I* and *K*), although social ability was unaffected (Fig. 6 *I* and *J*). In summary, reducing synaptic CD47 expression improved social novelty ability in 16p11.2 deletion mice.

## Discussion

The current study identified a significant link between CD47-mediated impairments in microglial phagocytosis and excitatory transmission, which contribute to cognitive and behavioral deficits in the widely used 16p11.2 deletion mouse model of ASD. Importantly, inhibiting or reducing CD47, using either a specific antibody or shRNA, markedly improved microglial synaptic pruning and alleviated social novelty deficits. These findings offer insights into the mechanisms underlying defective synaptic pruning by microglia, which ultimately leads to disruptions in excitatory transmission and the development of ASD-like behaviors.

Microglia, the resident immune cells of the brain, are responsible for phagocytosing dead cells and cellular debris during inflammation. Emerging evidence suggests that microglia also play a vital role in the brain’s normal functioning. Specifically, microglia preferentially engulf weaker or less active synapses, thereby promoting the refinement of neural circuits by preserving stronger and more active synapses. Although impaired microglial synaptic pruning has been linked to the development of ASD phenotypes (7, 8, 12, 25, 26), microglial changes have not been consistently demonstrated even within the same type of ASD animal model. For example, in the maternal immune activation (MIA) stress model, elevation in microglial numbers and levels of the proinflammatory cytokine IL-1β have been reported (27, 28). However, other studies reported reduced microglial reactivity in MIA stress mouse model, which affects long-term microglial function and disrupts proper striatal circuit development (26). The 16p11.2 deletion mouse model is commonly used for ASD studies. We previously performed transcriptomics analysis of the striatum of 16p11.2 deletion mice (29) and validated the reduced expression of several genes within the 16p11.2 deletion region, including Tbx6, Kif22, Maz, Asphd1, and others. In the present study, we found no increase in microglial numbers, surface area, or volume; however, we noted increased ramification compared to WT littermates, suggesting a more homeostatic state of microglia in the 16p11.2 deletion mice (30). Consequently, 16p11.2 deletion mice exhibited a significant decrease in microglial phagocytic capacity, increased number of synapses, and heightened excitatory transmission within the PFC. These alterations were associated with increased local field potential activity in the PFC, reduced long-range prefrontal connectivity (16), disrupted thalamic-prefrontal wiring, and decreased low-frequency neuronal synchronization (20).

An alternative mechanism to explain excessive spine formation in the ASD brain is that microglia may actively contribute to the development of synaptic spines, independent of their phagocytic activity. This process can lead to an increase in synaptic density. Under normal physiological conditions, microglia promote neuritogenesis by inducing filopodia formation and support spine maturation (31, 32). In the MIA mouse model, microglia increase the release of neuritogenic factors (33) while reducing the expression of synaptic pruning factors (34). Consequently, microglia in the MIA brain contribute to an increase in total spine density and filopodia formation (33). This alternative mechanism in the 16p11.2 deletion mice model warrants further investigation.

Overexpression of CD47 protects synapses from excess microglia-mediated pruning during development (14) and is associated with brain overgrowth in individuals with 16p11.2 deletion syndrome (17). Mice lacking CD47 exhibited increased microglial engulfment and reduced synapse numbers. CD47-deficient mice also displayed increased functional pruning, as measured by electrophysiology (13). The current western blotting and immunostaining findings demonstrated that CD47 expression was indeed up-regulated in 16p11.2 deletion mice. CD47 was coexpressed at the synapse, which was positive for VGLUT1 expression and was essential for activity-dependent changes in engulfment. Thus, the CD47 acts a “don’t eat me” signal overexpressed in the 16p11.2 deletion carriers and contributes to reduced phagocytosis in vivo.

Based on these observations, CD47 has been suggested as a valid target for treating ASD subtypes that have increased CD47 expression (17). We tested this hypothesis utilizing a CD47 antibody and rAAV CD47-shRNA. Both interventions were able to reverse deficits in microglial phagocytosis, improve excitatory transmission, and alleviate social novelty deficits in 16p11.2 deletion mice. However, despite these enhancements, impaired recognition memory persisted in these mice following treatment. This absence of an effect on recognition memory could potentially be attributed to the ineffectiveness of rAAV infection in the PFC. A future molecular genetics approach involving the offspring from crossing CD47 knockout mice with 16p11.2 deletion mice holds promise for elucidating this hypothesis. We anticipate that effective CD47 blockade will ameliorate social novelty and recognition memory deficits in 16p11.2 deletion mice. Nevertheless, the observation that LPS treatment effectively increased microglia phagocytic capacity, as shown in *SI Appendix*, Fig. S3. In addition, the responsiveness of microglia to LPS treatment in 16p11.2 deletion mice was similar to that in the WT mice, which suggests that LPS treatment may represent a therapeutic approach for ASD. Indeed, administering a low dose of LPS restored microglial synaptic pruning, normalized synaptic neurotransmission, and rescued behavioral impairments in an ASD mice model lacking Gls1 in CamKIIα-positive neurons (35).

Our study emphasizes the association of neuronal CD47 in facilitating microglial synaptic pruning, improving excitatory transmission, and reducing behavioral deficits in 16p11.2 deletion mice. This study constitutes the first indication that targeting CD47 shows potential as a therapeutic approach for enhancing outcomes in ASD. Further investigations are necessary to strengthen the role of CD47 interference as an effective therapeutic strategy for ASD.

A limitation of this mouse model is that it only reflects a small proportion (5.5%) of autism cases linked to genetic factors (5). To ascertain the broader implications of CD47 knockdown, future investigations should explore its effects on more prevalent murine models of autism, such as the BTBR mice model (36), the prenatal maternal immune activation stress models, such as the MIA and VPA models (27, 37). The comparative examination will be pivotal in gauging the clinical significance of CD47 intervention as a prospective therapy for ASD. Furthermore, synaptic pruning is essential for establishing precise neuronal circuitry during development and facilitating synaptic plasticity in the adult brain(13, 38). This process relies on a balance between “find me,” “eat me,” and “don’t eat me” signals (39, 40). In patients with 16p11.2 deletion syndrome, there is not only an increase in CD47 levels but also an upregulation of calreticulin, a prophagocytic eat me signal (17). Our current study on CD47 data underscores its importance and suggests that further research is needed to elucidate how this balance is regulated and dysregulated in the diseased brain.

## Materials and Methods

### Animals.

Mice carrying the 16p11.2 deletion on a hybrid C57BL/6N × 129 Sv genetic background were obtained from Jackson Laboratory and bred locally (JAX:013128). Both male and female mice with the 16p11.2 deletion aged 2 to 3 mo were selected for the study. Mice were housed in a controlled environment in a pathogen-free SPFII animal facility at 23 ± 1 °C and 50 ± 10% humidity and were subjected to a 12:12 h light/dark cycle (7 a.m. to 7 p.m.) with a light intensity of 15 to 20 lx during the light period, except during cleaning and experimental procedures when the room light was at 200 lx. Mice were group-housed in ventilated cages with six animals per cage, and food and water ad libitum were provided. Experimenters remained blinded to the animals’ treatments and sample processing throughout the experimentation and analysis.

### Ethical Approval and Animal Experimentation Design.

The Animal Care Committee of the Southern University of Science and Technology (Shenzhen, China) approved the animal experiment protocols. The ARRIVE guidelines for designing, performing, and reporting animal experimentation were followed (41). Mice in the study were randomly assigned to groups to ensure total randomization. Efforts were made to minimize animal numbers and suffering. Inclusion criteria were based on the identical age and sex of the mice. The AEEC Animal Experimentation Sample Size Calculator was utilized to determine the minimum sample size needed for the study hypothesis (42). Results indicated that a minimum of six mice per group were required for behavioral studies to achieve meaningful statistical differences. Additionally, at least three mice per group were used for slice electrophysiology, immunostaining, and the Golgi staining.

### Drug Administration.

LPS at a concentration of 0.02 mg/mL (19661, Cayman) was dissolved in saline, and intraperitoneal administrations of LPS at a dosage of 0.2 mg/kg were conducted for 7 d in WT and 16p11.2 deletion mice. On the 8th day, the mice brains were harvested. The anti-CD47 antibody at a concentration of 2 mg/mL (A2036, Selleck) was dissolved in aCSF solution, and 1 μL of the anti-CD47 antibody was locally injected into the bilateral prefrontal cortex (PFC) of 16p11.2 deletion mice once. In addition, 4 mg/mL anti-CD47 (A2036, Selleck) and 4 mg/mL F(ab’)_2_ (A24478, ThermoFisher) are mixed in equal volume. Once, 1 μL of the mixture of antibodies was locally injected into the bilateral PFC of 16p11.2 deletion mice. Three days later, the mice brains were collected. During the in vitro electrophysiology recording, the anti-CD47 antibody was diluted to a concentration of 2 μg/mL in aCSF. The brain slices of 16p11.2 deletion mice were immersed for 30 min, and the electrophysiology recording was initiated.

### Mouse Behavioral Tests.

#### Open field test.

The open-field test was conducted to evaluate locomotion and anxiety levels in mice, following a previously described method (43). Prior to the test, mice were allowed to acclimate to the testing room for 1 h. Each mouse was then placed in the center zone of the open field (40 × 40 × 40 cm), which was divided into 16 sections, with the four middle sections (20 cm × 20 cm) designated as the center area. The EthoVision XT software from Noldus Information Technology (Leesburg) was used to record the total distance traveled by the mice and the time spent in the center area during a 10-min period.

#### Y-maze test.

The Y-maze test was conducted to assess spontaneous alternations, reflecting working memory capacity. The maze consists of three arms with high walls and a triangular central platform (30 cm × 5 cm × 15 cm), each arm featuring visual cues at the end. Prior to the test, mice were given 1 h to explore the testing room. During the test, each mouse was placed on the central platform and allowed to move freely for 5 min. The sequence and total number of arms entered by each mouse were recorded using EthoVision XT software. Percentage alternations were then calculated using the formula: percentage alternations = number of three consecutive entries into a new arm/(total number of arms entered − 2).

#### NOR test.

The experiments were carried out in an open field box (40 cm × 40 cm × 40 cm) as previously described (44). Prior to testing, the mice were allowed to acclimate to the test room for 1 h. In the initial stage, two identical plastic toys were placed in the corners of the box, and the mice had 10 min to explore them. Subsequently, the mice were removed from the box and returned to their home cage. Two hours later, the mice were reintroduced to the test box, with one of the plastic toys replaced by a new toy of similar size but different color and shape. The mice were given 10 min to explore these objects. The time spent by the mouse’s nose tip within a square with a side length of 6 cm was measured, with exploration time automatically recorded by the EthoVision XT software.

#### Three-chamber test.

The methods used were as we previously described (16). The social behavior of mice was studied using a three-chamber apparatus that was divided into three interconnected chambers with transparent plexiglass. The mice were first habituated to the apparatus for 10 min. Sociability was then evaluated during a second 10-min period, during which the test mice could interact with either an empty cage or a genotype, age, and sex-matched stranger mouse (Mouse 1) that was placed in a cage in one of the chambers. Preference for social novelty was then assayed in a third 10-min period by introducing a second stranger mouse (Mouse 2) into the previously empty cage. The time spent interacting with the empty cage, Mouse 1, or Mouse 2 was recorded, and the statistical range of a circle with a diameter of 14 cm was measured using EthoVision XT 10 software. The sociability index (SI) and the social novelty preference index (SNI) were calculated as follows:

$$\mathrm{Sociability}\;\mathrm{index}=\frac{\mathrm{interaction}\;\mathrm{time}\;\mathrm{with}\;\mathrm{mouse}1-\mathrm{interaction}\;\mathrm{time}\;\mathrm{with}\;\mathrm{empty}\;\mathrm{cage}}{\mathrm{interaction}\;\mathrm{time}\;\mathrm{with}\;\mathrm{mouse}1+\mathrm{interaction}\;\mathrm{time}\;\mathrm{with}\;\mathrm{empty}\;\mathrm{cage}},$$

$$\text{Social}\;\text{novelty}\;\text{preference}\;\text{index}=\frac{\mathrm{interaction}\;\mathrm{time}\;\mathrm{with}\;\mathrm{mouse}2-\mathrm{interaction}\;\mathrm{time}\;\mathrm{with}\;\mathrm{mouse}1}{\mathrm{interaction}\;\mathrm{time}\;\mathrm{with}\;\mathrm{mouse}2+\mathrm{interaction}\;\mathrm{time}\;\mathrm{with}\;\mathrm{mouse}1}.$$

### Stereotaxic Surgery.

The methods used were as we described previously (45). The mice were anesthetized with isoflurane and then received bilateral injections of 250 nanoliters of AAV2/9-Syn-CD47 shRNA-ZsGreen (concentration at 1.9E+12 vg/mL; vg, viral genome) or AAV2/9-Syn-scrambled shRNA-ZsGreen (concentration at 2.2E+12 vg/mL) from HANBIO Technology (China), into the PFC at a rate of 0.05 microliters per minute. The injection site was targeted at AP: +1.98 mm, ML: ±0.3 mm, DV: −2.0 mm from bregma. The needle remained in place for 5 min postinjection before being removed. The mice were allowed to recover in their home cage until fully awake. To reduce pain, meloxicam (1 mg/kg, s.c.) and penicillin (3,000 U per mouse, i.p.) were administered once daily for 3 d. The AAV was expressed for 3 wk to label neurons with ZsGreen. The nucleotide sequence of the shRNA CD47 is 5′-CACCGAAGAAATGTTTGTGAA-3′, the nucleotide sequence of the scrambled shRNA is 5′-TTCTCCGAACGTGTCACGTAA-3′.

### Brain Slice Electrophysiology for sEPSC, PPR, and AMPA/NMDA Ratio.

The protocol for brain slice preparation was adapted from previous studies (44–46). Mice were anesthetized with 1% pentobarbital sodium and killed by decapitation. The mouse brain was dissected and immersed in ice-cold aCSF containing (in mM): 30 NaCl, 26 NaHCO_3_, 10 D-glucose, 4.5 KCl, 1.2 NaH_2_PO_4_, 1 MgCl_2_, 194 sucrose, and 1.5 HCl with pH at 7.4 and an osmolarity of 300 to 310 mOsm. Additionally, the solution was bubbled with 95% O_2_/5% CO_2_. Coronal brain slices (350 μm) were prepared using a vibratome (VT1120S, Leica Systems, Germany). The slices were allowed to recover for 30 min at 34 °C in aCSF containing (in mM): 124 NaCl, 26 NaHCO_3_, 10 D-glucose, 4.5 KCl, 1.2 NaH_2_PO_4_, 1 MgCl_2_, 2 CaCl_2_, 29.2 sucrose, and 1 HCl with pH at 7.4 and an osmolarity of 300 to 310 mOsm. Additionally, the solution was bubbled with 95% O_2_/5% CO_2_. Following transfer to a holding chamber at room temperature, recordings commenced only after at least 1 h of recovery. The slices were then positioned in a recording chamber (RC26G, Warner Instruments, USA) on the x-y stage of an upright microscope (BX51W; Olympus, Japan) and perfused with aCSF at a 2 mL/min rate. All recordings were conducted at room temperature.

The sEPSCs, PPR, and AMPA/NMDA ratio in pyramidal neurons were recorded using patch clamping techniques. The recording pipettes were filled with the following solution (in mM): 125 CsMeSO_3_, 5 NaCl, 10 HEPES (Na^+^ salt), 5 QX314, 1.1 EGTA, 4 ATP (Mg^2+^ salt), and 0.3 GTP (Na^+^ salt). sEPSCs of pyramidal neurons located in the PFC were recorded in aCSF supplemented with 20 μM bicuculline at a holding potential of −60 mV. The PPR was recorded to evaluate the probability of presynaptic glutamate release. Electrical stimulation (0.1 ms square pulse) was administered using a glass electrode filled with aCSF, positioned within 0.1 mm of the recording site. Throughout the recording session, aCSF containing 20 μM bicuculline was continuously perfused. PPR recordings were conducted using two stimulations with durations of 20 ms, 50 ms, 100 ms, and 200 ms. To obtain the NMDAR–EPSC-to-AMPAR– EPSC ratio (AMPA/NMDA ratio), AMPAR–EPSC was first recorded at −60 mV. Then, the mixture of AMPAR–EPSC and NMDAR–EPSC was recorded at +40 mV with the same stimulation pulse (0.1 ms). The peak of NMDAR–EPSC was calculated at 50 ms from the onset of the EPSC. Throughout the recording session, aCSF containing 20 μM bicuculline was continuously perfused. The acquisition frequency was set at 20.0 kHz, and the filter was adjusted to 2.9 kHz. sEPSCs were analyzed using Mini-analysis (Synaptosoft Inc.), focusing on frequency and amplitude. Additionally, PPR, AMPAR–EPSC and NMDAR–EPSC traces were imported into Fitmaster software (HEKA Elektronik) to measure evoked EPSC amplitude.

### Immunofluorescence Staining.

Mice were anesthetized with phenobarbital sodium salt (0.1 g/kg) and perfused transcardially with ice-cold 0.01 M PBS, followed by 4% paraformaldehyde (PFA) in 0.01 M PBS. The brains were fixed in 4% PFA for 2 d, then dehydrated in 15% and 30% sucrose, respectively. Coronal sections (30 μm) were prepared and blocked with a solution containing PBS, 0.3% Triton X-100, and 10% goat serum for 1 h at room temperature. The sections were then incubated overnight at 4 °C with primary antibodies: anti-IBA1 (rabbit, 1:500, ab178847, Abcam), anti-VGLUT1 (guinea pig, 1:500, 135304, Synaptic Systems), anti-CD68 (rat, 1:500, MCA1957, Bio-Rad), anti-PV (rabbit, 1:1,000, ab11427, Abcam), anti-NeuN (mouse, 1:500, ab104224, Abcam), anti-CD47 (rabbit, 1:200, ab214453, Abcam), anti-CD47(rat, 1:200, 555297, BD Pharmingen™). After primary antibody incubation, sections were treated with appropriate secondary antibodies for 1 h at room temperature. Visualization was performed using a Zeiss LSM980 confocal microscope with a 20× or 63× lens at the imaging resolution of 1,024 * 1,024 pixels.

### Analysis of 3D Microglia Engulfment and Microglia Morphology.

The method used to analyze microglial morphology was precisely as we previously described (16). The z-stack images (at 1 μm intervals) were captured using a Zeiss LSM980 confocal microscope with a 63× lens. Microglia were selected randomly based on positive staining for IBA1, ensuring an unbiased approach. Individual microglia, including those with VGLUT1 signaling, were isolated using Image J and transferred to Imaris 9.0.0 software (Oxford Instruments, USA). Initially, 3D volume surface renderings of the microglial channels were generated to compute each microglial cell’s volume and surface area. Subsequently, a new channel representing engulfed VGLUT1 was established using the mask function to determine the volume of engulfed VGLUT1. The engulfment percentage was calculated as the volume of internalized VGLUT1 puncta divided by the volume of the microglial cell (14, 47). Next, using the filament tool, individual microglia were traced using the default settings [autopath (no loops) and cell body sphere region ~10 μm] and manually modified if necessary, and filament dendrite length of microglia was measured automatically (48).

### The Golgi Staining.

The Golgi staining was conducted using the FD Rapid GolgiStain™ kit (FD NeuroTechnologies, Ellicott City, MD) following the manufacturer’s instructions and as previously described (49). Briefly, the brains were rinsed with double distilled water (ddH_2_O) and then immersed in a 1:1 mixture of FD Impregnation Solution A and B in darkness for 3 wk at room temperature. The solution was replaced once every 24 h. Following impregnation, the brains were transferred to FD Solution C and stored in darkness for 5 d, with the solution being replaced after 24 h.

The individual brains were mounted on a specimen disc with optimum cutting temperature compound and subjected to snap freezing and cryosectioning on a Leica CM1950. Coronal sections of 200 µm thickness were then cut and transferred to agar-treated slides for staining. After drying for 4 d, the brain sections were rinsed twice with ddH_2_O and stained in a mixture of FD Solution D, FD Solution E, and ddH_2_O in a 1:1:2 v/v ratio for 10 min. The stained sections were rinsed twice with ddH_2_O for 4 min each and sequentially dehydrated in 50%, 75%, 95%, and 4 times in 100% ethanol, with each dehydration step lasting 4 min. Following dehydration, the sections were cleared 3 times in xylene for 4 min each rinse and sealed with a resinous mounting medium. The Golgi-stained sections were examined under an optical microscope a 100× lens at the imaging resolution of 1,024 * 1,024 pixels, and the images were analyzed using Image J software to determine the density per 10 µm of dendritic length.

### Western Blotting.

The method used was essentially as we previously described (46). Briefly, the prefrontal cortex was removed and lysed in 200 μL lysis buffer (Cat. #C500008, Sangon Biotech, China) containing proteinase and phosphatase inhibitors. The lysates were centrifuged at 13,680 g for 10 min at 4 °C, and the supernatant was collected and boiled in boiling water for 10 min with a loading buffer (4:1 ratio). Whole proteins were electrophoresed in a 10% SDS-PAGE gel and then transferred to polyvinylidene fluoride (PVDF) membranes (0.45 μm) using iBlot 2 Dry Blotting System (Thermo Fisher Scientific Systems, USA). After blocking in 5% skim milk, the PVDF membranes were incubated with the primary antibody (anti-IBA1: rabbit, 1:1,000, ab178847; anti-CD47: rabbit, 1:1,000, ab214453; anti-CORO1A, rabbit, 1:1,000, ab228635; anti-GAPDH, rabbit, 1:3,000, ab181602; anti-β-ACTIN, mouse, 1:3,000, ab6276) overnight at 4 °C. The PVDF membranes were then incubated with a secondary antibody after washing three times with TBST. Protein band intensities were detected with Tanon (Shanghai, China).

### Statistical Analysis.

All data were expressed as the mean ± SEM. Statistical analyses were conducted using Prism (V9, GraphPad Software, USA). Data distribution was assessed initially using the Shapiro–Wilk test to determine suitability for parametric or nonparametric tests. An unpaired *t* test was employed for normally distributed data in two-group comparisons. Nonnormally distributed data were analyzed using the Mann–Whitney *U* test. The Kruskal–Wallis test with Dunn’s multiple comparisons post hoc test was employed for nonnormally distributed data in three-group comparisons. The paired-pulse ratio was evaluated using two-way repeated measures ANOVA with Sidak’s post hoc tests. Specific experiment details, including exact sample sizes (n), precision measures, statistical tests performed, and definitions of significance, are provided in figure legends. Statistical significance was set at *P* < 0.05.

## Supplementary Material

Appendix 01 (PDF)

## Data Availability

All study data are included in the article and/or *SI Appendix*.

## References

[1] D. K. Sokol, M. Edwards-Brown, Neuroimaging in autistic spectrum disorder (ASD). J. Neuroimaging **14**, 8–15 (2004). 14748203

[2] S. R. Sharma, X. Gonda, F. I. Tarazi, Autism spectrum disorder: Classification, diagnosis and therapy. Pharmacol. Ther. **190**, 91–104 (2018). 29763648 10.1016/j.pharmthera.2018.05.007

[3] A. Masi, M. M. DeMayo, N. Glozier, A. J. Guastella, An overview of autism spectrum disorder, heterogeneity and treatment options. Neurosci. Bull. **33**, 183–193 (2017). 28213805 10.1007/s12264-017-0100-y PMC5360849

[4] M. Yang, F. C. Lewis, M. S. Sarvi, G. M. Foley, J. N. Crawley, 16p11.2 Deletion mice display cognitive deficits in touchscreen learning and novelty recognition tasks. Learn. Mem. **22**, 622–632 (2015). 26572653 10.1101/lm.039602.115 PMC4749736

[5] B. Rein, Z. Yan, 16p11.2 copy number variations and neurodevelopmental disorders. Trends Neurosci. **43**, 886–901 (2020). 32993859 10.1016/j.tins.2020.09.001 PMC7606557

[6] C. A. Pardo, D. L. Vargas, A. W. Zimmerman, Immunity, neuroglia and neuroinflammation in autism. Int. Rev. Psychiatry **17**, 485–495 (2005). 16401547 10.1080/02646830500381930

[7] J. I. Rodriguez, J. K. Kern, Evidence of microglial activation in autism and its possible role in brain underconnectivity. Neuron Glia Biol. **7**, 205–213 (2011). 22874006 10.1017/S1740925X12000142 PMC3523548

[8] K. Suzuki , Microglial activation in young adults with autism spectrum disorder. JAMA Psychiatry **70**, 49–58 (2013). 23404112 10.1001/jamapsychiatry.2013.272

[9] J. R. Guedes, P. A. Ferreira, J. M. Costa, A. L. Cardoso, J. Peça, Microglia-dependent remodeling of neuronal circuits. J. Neurochem. **163**, 74–93 (2022). 35950924 10.1111/jnc.15689 PMC9826178

[10] R. C. Paolicelli , Synaptic pruning by microglia is necessary for normal brain development. Science **333**, 1456–1458 (2011). 21778362 10.1126/science.1202529

[11] A. Mordelt, L. D. de Witte, Microglia-mediated synaptic pruning as a key deficit in neurodevelopmental disorders: Hype or hope? Curr. Opin. Neurobiol. **79**, 102674 (2023). 36657237 10.1016/j.conb.2022.102674

[12] R. C. Paolicelli, C. T. Gross, Microglia in development: Linking brain wiring to brain environment. Neuron Glia Biol. **7**, 77–83 (2011). 22857738 10.1017/S1740925X12000105

[13] E. K. Lehrman , CD47 protects synapses from excess microglia-mediated pruning during development. Neuron **100**, 120–134.e6 (2018). 30308165 10.1016/j.neuron.2018.09.017 PMC6314207

[14] X. Ding , Loss of microglial SIRPalpha promotes synaptic pruning in preclinical models of neurodegeneration. Nat. Commun. **12**, 2030 (2021). 33795678 10.1038/s41467-021-22301-1 PMC8016980

[15] B. Wamsley , Molecular cascades and cell type–specific signatures in ASD revealed by single-cell genomics. Science **384**, eadh2602 (2024). 38781372 10.1126/science.adh2602

[16] J. Ju , Adenosine mediates the amelioration of social novelty deficits during rhythmic light treatment of 16p11.2 deletion female mice. Mol. Psychiatry **29**, 3381–3394 (2024), 10.1038/s41380-024-02596-4. 38740879 PMC11541200

[17] J. Li , Overexpression of CD47 is associated with brain overgrowth and 16p11.2 deletion syndrome. Proc. Natl. Acad. Sci. U.S.A. **118**, e2005483118 (2021). 33833053 10.1073/pnas.2005483118 PMC8053942

[18] M. Sundberg , 16p11.2 deletion is associated with hyperactivation of human iPSC-derived dopaminergic neuron networks and is rescued by RHOA inhibition in vitro. Nat. Commun. **12**, 2897 (2021). 34006844 10.1038/s41467-021-23113-z PMC8131375

[19] G. Elberg , Deletion of SIRPalpha (signal regulatory protein-alpha) promotes phagocytic clearance of myelin debris in Wallerian degeneration, axon regeneration, and recovery from nerve injury. J. Neuroinflammation **16**, 277 (2019). 31883525 10.1186/s12974-019-1679-x PMC6935070

[20] A. Bertero , Autism-associated 16p11.2 microdeletion impairs prefrontal functional connectivity in mouse and human. Brain **141**, 2055–2065 (2018). 29722793 10.1093/brain/awy111

[21] J. Pucilowska , The 16p11.2 deletion mouse model of autism exhibits altered cortical progenitor proliferation and brain cytoarchitecture linked to the ERK MAPK pathway. J. Neurosci. **35**, 3190–3200 (2015). 25698753 10.1523/JNEUROSCI.4864-13.2015 PMC6605601

[22] W. Wang , Chemogenetic activation of prefrontal cortex rescues synaptic and behavioral deficits in a mouse model of 16p11.2 deletion syndrome. J. Neurosci. **38**, 5939–5948 (2018). 29853627 10.1523/JNEUROSCI.0149-18.2018 PMC6021990

[23] Q. Q. Han, S. Y. Shen, L. F. Liang, X. R. Chen, J. Yu, Complement C1q/C3-CR3 signaling pathway mediates abnormal microglial phagocytosis of synapses in a mouse model of depression. Brain Behav. Immun. **119**, 454–464 (2024). 38642614 10.1016/j.bbi.2024.04.018

[24] X. Wu , Complement C1q drives microglia-dependent synaptic loss and cognitive impairments in a mouse model of lipopolysaccharide-induced neuroinflammation. Neuropharmacology **237**, 109646 (2023). 37356797 10.1016/j.neuropharm.2023.109646

[25] J. Wu , Microglial over-pruning of synapses during development in autism-associated SCN2A-deficient mice and human cerebral organoids. Mol. Psychiatry **29**, 2424–2437 (2024), 10.1038/s41380-024-02518-4. 38499656

[26] L. N. Hayes , Prenatal immune stress blunts microglia reactivity, impairing neurocircuitry. Nature **610**, 327–334 (2022). 36171283 10.1038/s41586-022-05274-z PMC13436803

[27] S. Yan, L. Wang, J. N. Samsom, D. Ujic, F. Liu, PolyI: C maternal immune activation on E9.5 causes the deregulation of microglia and the complement system in mice, leading to decreased synaptic spine density. Int. J. Mol. Sci. **25**, 5480 (2024). 38791517 10.3390/ijms25105480 PMC11121703

[28] C. G. J. Guerrin , Prenatal infection and adolescent social adversity affect microglia, synaptic density, and behavior in male rats. Neurobiol. Stress **27**, 100580 (2023). 37920548 10.1016/j.ynstr.2023.100580 PMC10618826

[29] J. Ju , Structural and lipidomic alterations of striatal myelin in 16p11.2 deletion mouse model of autism spectrum disorder. Front. Cell Neurosci. **15**, 718720 (2021). 34483844 10.3389/fncel.2021.718720 PMC8416256

[30] R. C. Paolicelli , Microglia states and nomenclature: A field at its crossroads. Neuron **110**, 3458–3483 (2022). 36327895 10.1016/j.neuron.2022.10.020 PMC9999291

[31] A. Miyamoto , Microglia contact induces synapse formation in developing somatosensory cortex. Nat. Commun. **7**, 12540 (2016). 27558646 10.1038/ncomms12540 PMC5007295

[32] L. Weinhard , Microglia remodel synapses by presynaptic trogocytosis and spine head filopodia induction. Nat. Commun. **9**, 1228 (2018). 29581545 10.1038/s41467-018-03566-5 PMC5964317

[33] S. Ikezu , Inhibition of colony stimulating factor 1 receptor corrects maternal inflammation-induced microglial and synaptic dysfunction and behavioral abnormalities. Mol. Psychiatry **26**, 1808–1831 (2021). 32071385 10.1038/s41380-020-0671-2 PMC7431382

[34] L. Fernandez de Cossio, A. Guzman, S. van der Veldt, G. N. Luheshi, Prenatal infection leads to ASD-like behavior and altered synaptic pruning in the mouse offspring. Brain Behav. Immun. **63**, 88–98 (2017). 27697456 10.1016/j.bbi.2016.09.028

[35] C. Ji , Glutaminase 1 deficiency confined in forebrain neurons causes autism spectrum disorder-like behaviors. Cell Rep. **42**, 112712 (2023). 37384529 10.1016/j.celrep.2023.112712

[36] L. Zheng , Human-derived fecal microbiota transplantation alleviates social deficits of the BTBR mouse model of autism through a potential mechanism involving vitamin B(6) metabolism. mSystems **9**, e0025724 (2024), 10.1128/msystems.00257-24. 38780265 PMC11237617

[37] N. Prince , Prebiotic diet normalizes aberrant immune and behavioral phenotypes in a mouse model of autism spectrum disorder. Acta Pharmacol. Sin. **45**, 1591–1603 (2024), 10.1038/s41401-024-01268-x. 38589690 PMC11272935

[38] S. Hong, L. Dissing-Olesen, B. Stevens, New insights on the role of microglia in synaptic pruning in health and disease. Curr. Opin. Neurobiol. **36**, 128–134 (2016). 26745839 10.1016/j.conb.2015.12.004 PMC5479435

[39] J. Cornell, S. Salinas, H. Y. Huang, M. Zhou, Microglia regulation of synaptic plasticity and learning and memory. Neural Regen. Res. **17**, 705–716 (2022). 34472455 10.4103/1673-5374.322423 PMC8530121

[40] A. Huo , Molecular mechanisms underlying microglial sensing and phagocytosis in synaptic pruning. Neural Regen. Res. **19**, 1284–1290 (2024). 37905877 10.4103/1673-5374.385854 PMC11467947

[41] N. Percie du Sert , The ARRIVE guidelines 2.0: Updated guidelines for reporting animal research. PLoS Biol. **18**, e3000410 (2020). 32663219 10.1371/journal.pbio.3000410 PMC7360023

[42] D. A. Fitts, Ethics and animal numbers: Informal analyses, uncertain sample sizes, inefficient replications, and type I errors. J. Am. Assoc. Lab. Anim. Sci. **50**, 445–453 (2011). 21838970 PMC3148647

[43] O. Leiter , Selenium mediates exercise-induced adult neurogenesis and reverses learning deficits induced by hippocampal injury and aging. Cell Metab. **34**, 408–423.e8 (2022). 35120590 10.1016/j.cmet.2022.01.005

[44] J. Ju, L. Liu, Y. Zhang, Q. Zhou, Effect of age onset on schizophrenia-like phenotypes and underlying mechanisms in model mice. Prog. Neuropsychopharmacol. Biol. Psychiatry **89**, 465–474 (2019). 30025793 10.1016/j.pnpbp.2018.07.015

[45] S. Zhang , Hypothermia evoked by stimulation of medial preoptic nucleus protects the brain in a mouse model of ischaemia. Nat. Commun. **13**, 6890 (2022). 36371436 10.1038/s41467-022-34735-2 PMC9653397

[46] L. Zheng , Rhythmic light flicker rescues hippocampal low gamma and protects ischemic neurons by enhancing presynaptic plasticity. Nat. Commun. **11**, 3012 (2020). 32541656 10.1038/s41467-020-16826-0 PMC7296037

[47] D. P. Schafer, E. K. Lehrman, C. T. Heller, B. Stevens, An engulfment assay: A protocol to assess interactions between CNS phagocytes and neurons. J. Vis. Exp. **88**, e51482 (2014), 10.3791/51482. PMC4188069 24962472

[48] M. J. Rigby , Increased expression of SLC25A1/CIC causes an autistic-like phenotype with altered neuron morphology. Brain **145**, 500–516 (2022). 35203088 10.1093/brain/awab295 PMC9014753

[49] M. Yu , Gallic acid disruption of Abeta(1–42) aggregation rescues cognitive decline of APP/PS1 double transgenic mouse. Neurobiol. Dis. **124**, 67–80 (2019). 30447302 10.1016/j.nbd.2018.11.009

