# Supplementary material for: The “don’t eat me” signal CD47 is associated with microglial phagocytosis defects and autism-like behaviors in 16p11.2 deletion mice
Source: Proc Natl Acad Sci U S A. 2025 Apr 16;122(16):e2411080122. doi: 10.1073/pnas.2411080122 (PMC12036979; doi:10.1073/pnas.2411080122)
Supplement: Supplementary file 1 — Appendix 01 (PDF) [file pnas.2411080122.sapp.pdf]

PNAS

Supporting Information for

The "don't eat me" signal CD47 is associated with microglial phagocytosis defects and autism-like behaviors in 16p11.2 deletion mice

Jun Ju[1], Yifan Pan[1], Xinyi Yang[1], Xuanyi Li[1], Jinghong Chen[1], Shiyu Wu[1], Sheng-Tao Hou[1,*]

1 Brain Research Centre, Department of Biology, School of Life Sciences, Southern University of Science and Technology, 1088 Xueyuan Blvd, Nanshan District, Shenzhen, 518055, Guangdong, P. R. China.

* Prof. Sheng-Tao Hou, Ph.D.

Email: hou.st@sustech.edu.cn

This PDF file includes:

Supporting text
Figures S1 to S8
Legends for Figures S1 to S8

Supporting Information Text

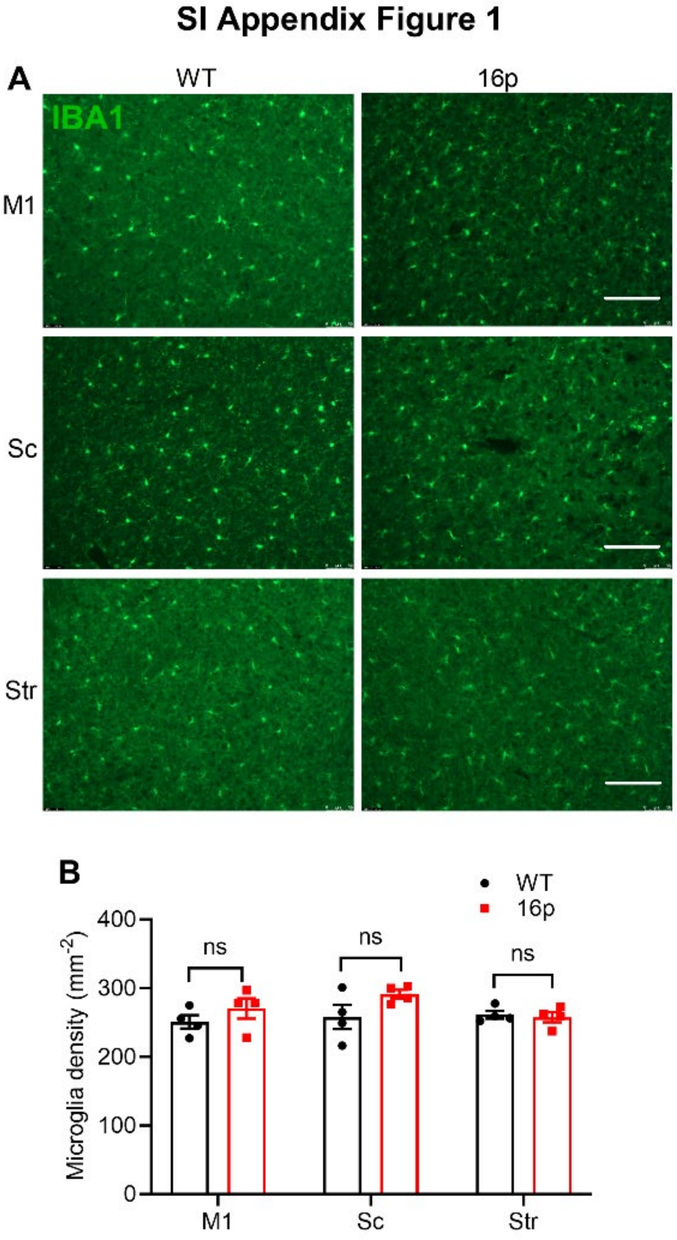

Supplementary Figure 1. Unaltered microglial numbers in different brain regions in 16p11.2 deletion mice

(A) Representative image of IBA1 microglia immunostaining in the primary motor cortex (M1), sensory cortex (Sc), and striatum (Str) in two mice groups. The scale bar: 100 μm. *(B)* Quantification of microglia intensity. The number of mice: WT n = 4 mice, 16p n = 4 mice; ns, not significant, unpaired t test.

SI Appendix Figure 2

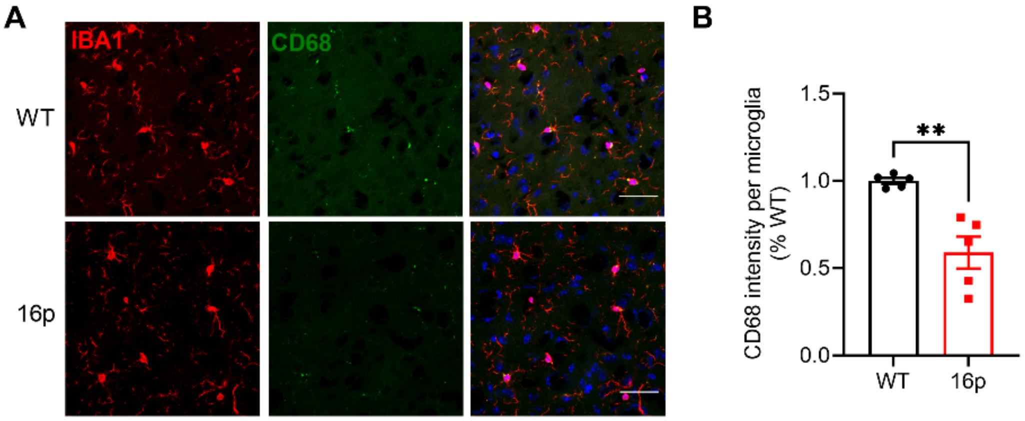

Supplementary Figure 2. Reduced CD68 expression in microglia in 16p11.2 deletion mouse PFC

(*A*) Representative images of IBA1 and CD68 co-immunostaining in the PFC. The scale bar: 100 μm. (*B*) Quantification of CD68 intensity per microglia (WT: *n* = 5 mice, 16p: *n* = 5 mice). **$P < 0.01$, unpaired *t* test.

SI Appendix Figure 3

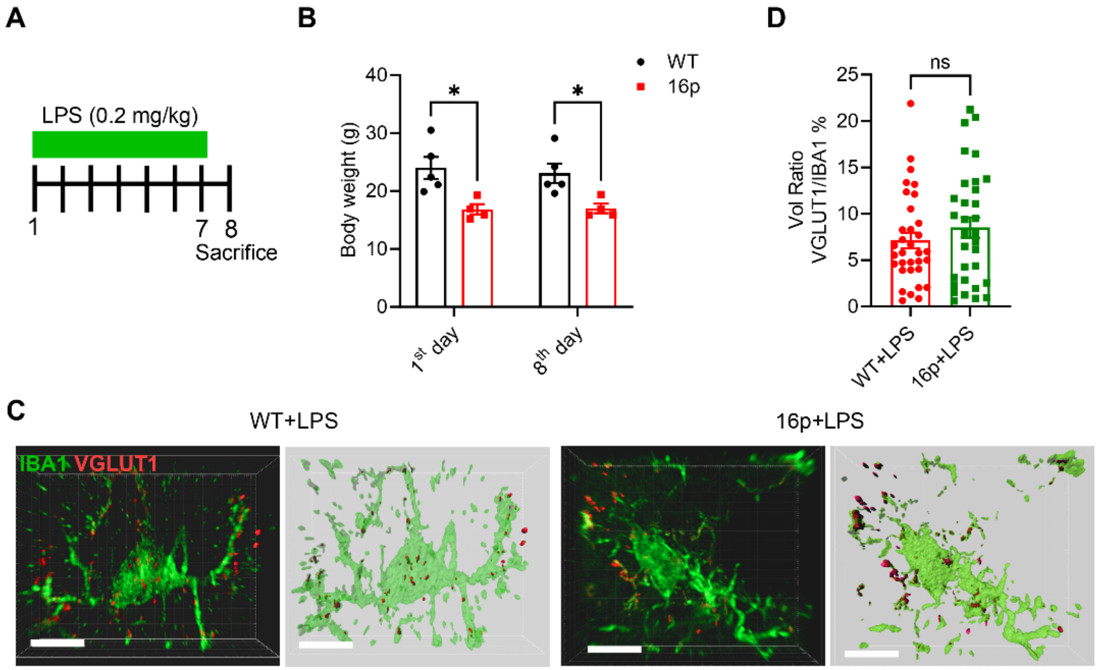

Supplementary Figure 3. LPS promoted microglia-dependent synapse pruning in both WT and 16p11.2 deletion mice

(*A*) The experimental scheme of LPS treatment (0.2 mg/kg). (*B*) The body weight of WT and 16p mice before and after LPS treatment (WT+LPS: *n* = 5 mice, 16p+LPS: *n* = 4 mice). (*C*) Representative images of IBA1 and VGLUT1 co-immunostaining in PFC. The scale bar: 10 μm. (*D*) Quantification of VGLUT1/IBA1 volume ratios in microglia (WT+LPS: *n* = 33 cells from 5 mice, 16p+LPS: *n* = 32 cells from 4 mice). ns, not significant, *P < 0.05, two-way RM ANOVA with Sidak's multiple comparisons post hoc test for panel B and Mann-Whitney U test for panel D.

SI Appendix Figure 4

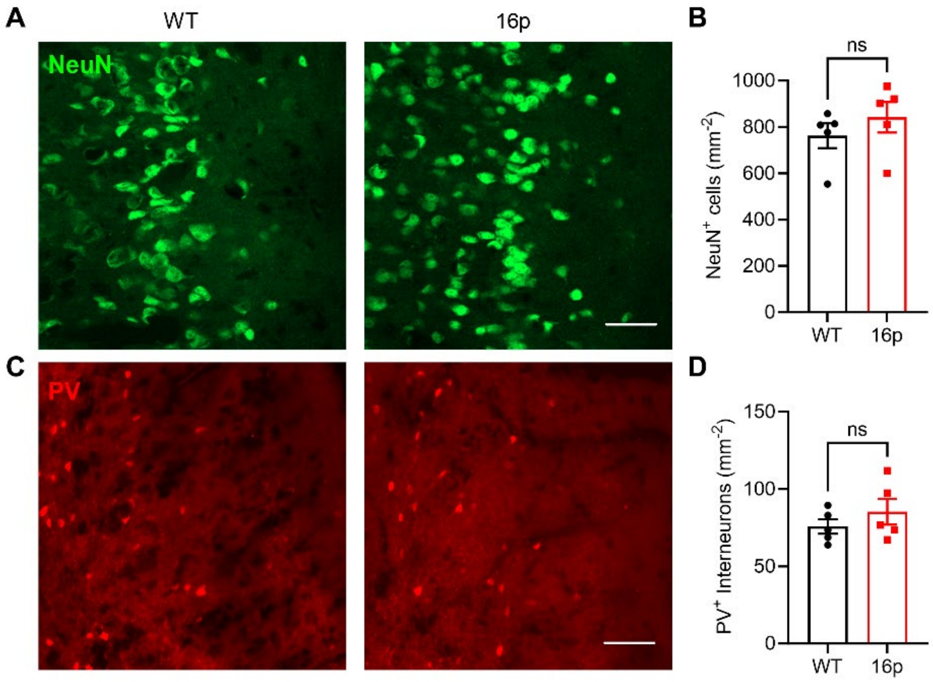

Supplementary Figure 4. Unaltered numbers of mature neurons and PV interneurons in 16p11.2 deletion mouse PFC

(*A*) Representative images of PFC immunostained with a NeuN antibody in two groups of mice. The scale bar: 100 µm. (*B*) Quantification of the density of $NeuN^+$ cells in the PFC (WT: *n* = 5 mice, 16p: *n* = 5 mice). (*C*) Representative image of PV interneurons in two groups of mice. The scale bar: 100 µm. (*D*) Quantification of the density of PV^+ interneurons in the PFC (WT: *n* = 5 mice, 16p: *n* = 5 mice). ns, not significant, unpaired *t* test.

SI Appendix Figure 5

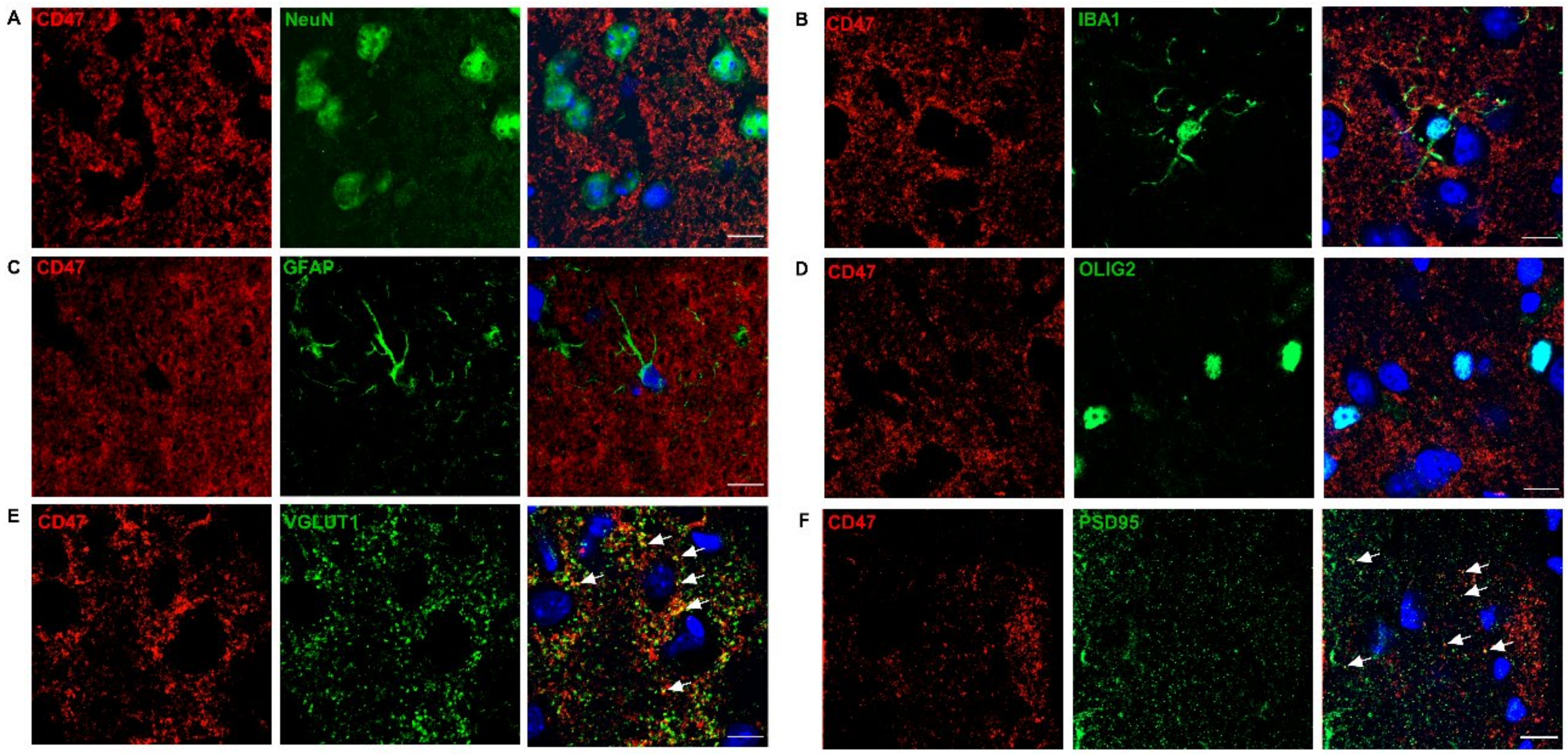

Supplementary Figure 5. The expression patterns of CD47 in the PFC of WT mice

(*A*) Representative images of CD47 and NeuN co-immunostaining in the PFC. The scale bar: 10 μm. (*B*) Representative images of CD47 and IBA1 co-immunostaining in the PFC. The scale bar: 10 μm. (*C*) Representative images of CD47 and GFAP co-immunostaining in the PFC. The scale bar: 10 μm. (*D*) Representative images of CD47 and OLIG2 co-immunostaining in the PFC. The scale bar: 10 μm. (*E*) Representative images of CD47 and VGLUT1 co-immunostaining in the PFC. The scale bar: 10 μm. (*F*) Representative images of CD47 and PSD95 co-immunostaining in the PFC. The scale bar: 10 μm. Rat-CD47 was used in this experiment.

SI Appendix Figure 6

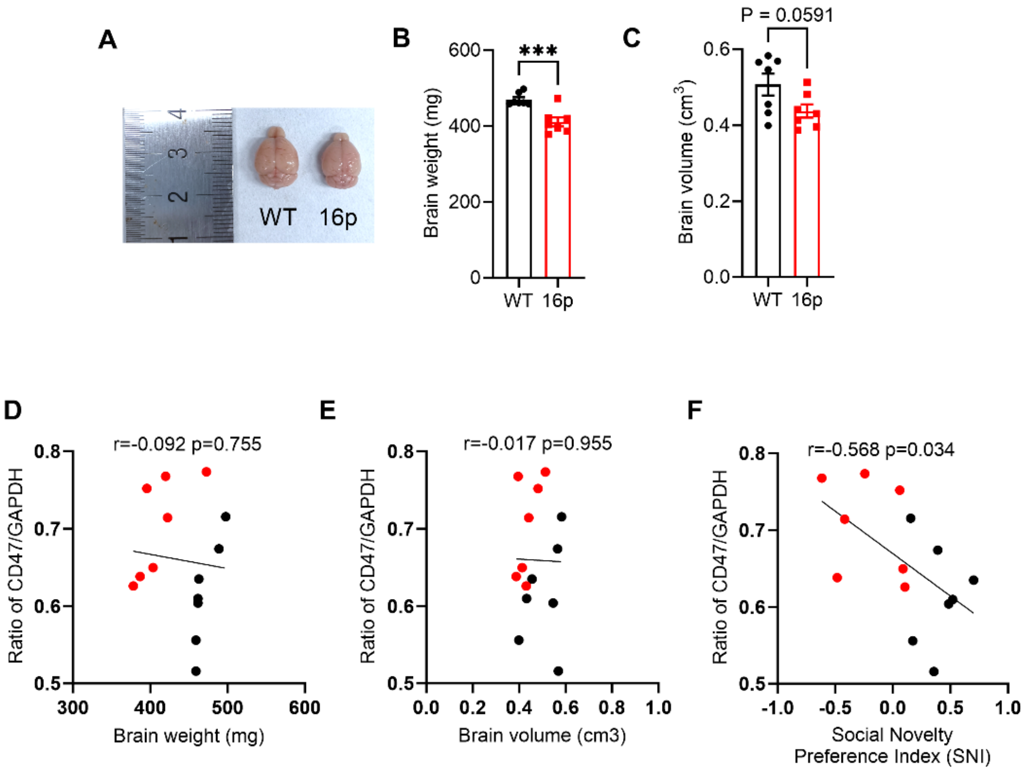

Supplementary Figure 6. The social novelty preference index (SNI) has a significant negative correlation with CD47 expression levels

(*A*) Representative images of brain images in WT and 16p11.2 deletion mice. (*B*) Quantification of the brain weight (WT: n = 7 mice, 16p: n = 7 mice). (*C*) Quantification of the brain volume (WT: n = 7 mice, 16p: n = 7 mice). Correlation analysis between CD47 expression levels with brain weight (*D*), brain volume (*E*) and social novelty preference index (*F*) (WT: n = 7 mice, 16p: n = 7 mice). ***P < 0.001, unpaired *t* test for panels B, C and simple linear regression for panels D, E, F.

SI Appendix Figure 7

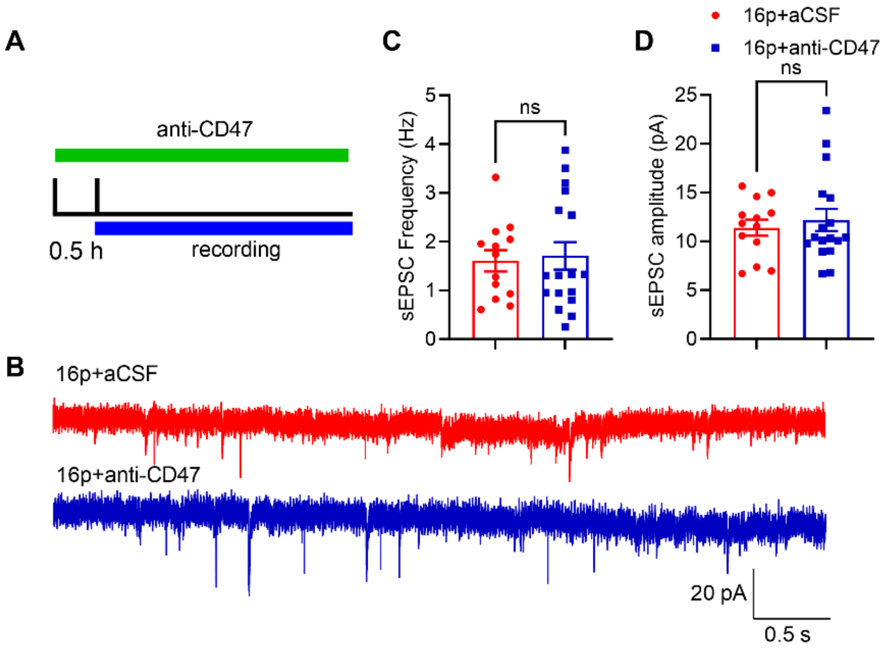

Supplementary Figure 7. Blocking PFC CD47 using a specific antibody did not affect excitatory transmission in 16p11.2 deletion mice brain slice

(*A*) The experimental scheme of anti-CD47 antibody treatment (2 μg/ml). (*B*) Representative traces of sEPSC recorded in PFC in two groups of mice. The scale bar: 20 pA/0.5 s. (*C*) Quantification of sEPSC frequency. (*D*) Quantification of sEPSC amplitude (16p+aCSF: *n* = 13 cells from 3 mice, 16p+anti-CD47: *n* = 17 cells from 3 mice). ns, not significant, Mann-Whitney U test.

SI Appendix Figure 8

A

B

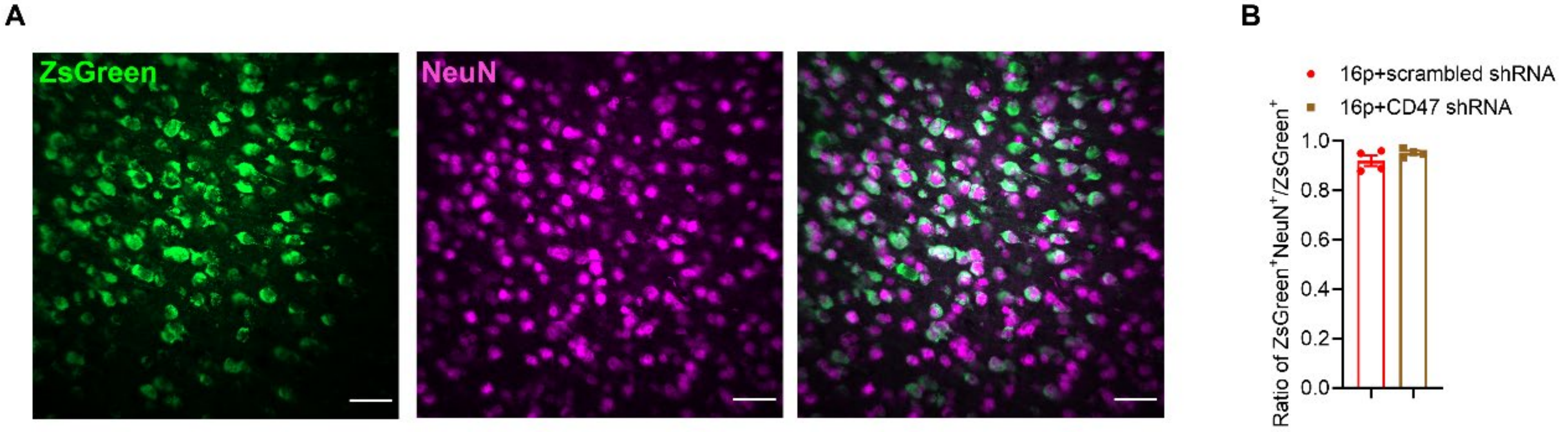

Supplementary Figure 8. The shRNA virus labels over 90% of neurons in the PFC

(*A*) Representative images of ZsGreen and NeuN co-immunostaining in the PFC. The scale bar: 50 μm. (*B*) Quantification of the ratio of $ZsGreen^{+}NeuN^{+}$ cells in $ZsGreen^{+}$ cells in the PFC (16p+scrambled shRNA: *n* = 5 mice, 16p+CD47 shRNA: *n* = 5 mice).
